# Recent Advancements in Microneedle Technology for Multifaceted Biomedical Applications

**DOI:** 10.3390/pharmaceutics14051097

**Published:** 2022-05-20

**Authors:** Deepak Kulkarni, Fouad Damiri, Satish Rojekar, Mehrukh Zehravi, Sarker Ramproshad, Dipali Dhoke, Shubham Musale, Ashiya A. Mulani, Pranav Modak, Roshani Paradhi, Jyotsna Vitore, Md. Habibur Rahman, Mohammed Berrada, Prabhanjan S. Giram, Simona Cavalu

**Affiliations:** 1 Department of Pharmaceutics, Srinath College of Pharmacy, Bajajnagar, Aurangabad 431136, India; deepakkulkarni68@gmail.com; 2 Laboratory of Biomolecules and Organic Synthesis (BIOSYNTHO), Department of Chemistry, Faculty of Sciences Ben M’Sick, University Hassan II of Casablanca, Casablanca 20000, Morocco; fouad.damiri@outlook.fr (F.D.); berrada_moh@hotmail.com (M.B.); 3 Department of Pharmaceutical Sciences and Technology, Institute of Chemical Technology, Mumbai 400019, India; rojekarsatish@gmail.com; 4 Departments of Medicine and Pharmacological Sciences, Icahn School of Medicine at Mount Sinai, New York, NY 10029, USA; 5 Department of Clinical Pharmacy Girls Section, Prince Sattam Bin Abdul Aziz University, Alkharj 11942, Saudi Arabia; mahrukh.zehravi@hotmail.com; 6 Department of Pharmacy, Ranada Prasad Shaha University, Narayanganj 1400, Bangladesh; ramproshad131135@gmail.com; 7 Department of Pharmaceutical Sciences, Rashtrasant Tukadoji Maharaj Nagpur University, Nagpur 440033, India; dipalidhoke7@gmail.com; 8 Department of Pharmaceutics, Dr. DY Patil Institute of Pharmaceutical Sciences and Research, Pimpri, Pune 411018, India; shubhammusale1010@gmail.com (S.M.); ashiyaayubmulani@gmail.com (A.A.M.); pranavmodak0079@gmail.com (P.M.); roshanisparadhi@gmail.com (R.P.); 9 National Institute of Pharmaceutical Education and Research, Ahmedabad 160062, India; vitorejyotsna@gmail.com; 10 Department of Global Medical Science, Wonju College of Medicine, Yonsei University, Wonju 26426, Korea; 11 Department of Pharmaceutical Sciences, University at Buffalo, The State University of New York, Buffalo, NY 14260, USA; 12 Faculty of Medicine and Pharmacy, University of Oradea, P-ta 1 Decembrie 10, 410087 Oradea, Romania

**Keywords:** MNs, drug delivery, nanoparticles, permeation, skin, transdermal

## Abstract

Microneedle (MNs) technology is a recent advancement in biomedical science across the globe. The current limitations of drug delivery, like poor absorption, low bioavailability, inadequate skin permeation, and poor biodistribution, can be overcome by MN-based drug delivery. Nanotechnology made significant changes in fabrication techniques for microneedles (MNs) and design shifted from conventional to novel, using various types of natural and synthetic materials and their combinations. Nowadays, MNs technology has gained popularity worldwide in biomedical research and drug delivery technology due to its multifaceted and broad-spectrum applications. This review broadly discusses MN’s types, fabrication methods, composition, characterization, applications, recent advancements, and global intellectual scenarios.

## 1. Introduction

Oral delivery is the most accepted route of administration for the treatment of disease, diagnosis, treatment, and the most widely studied topic by formulation scientists during the first and second generations of drug delivery. Oral drug delivery has the disadvantages of systemic metabolism, poor absorption, and a lack of tissue selectivity, leading to decreased therapeutic effects [1]. This necessitates the development of other dosage forms such as parenteral, transdermal, intravesical and other novel drug delivery systems. Ancient medicines for therapeutic effect applied on skin surface area, for various types of skin disease, wound healing activity, and cosmetic dermatological applications. However, the skin has an impermeable barrier for efficient and targeted delivery [2]. Scientists developed syringes and needles for local drug delivery applications to overcome this limitation. Although, these developed systems also have some disadvantages, such as poor patient compliance due to the pain in administration and the invasive nature that requires trained medical practitioners. Furthermore, with the advent of polymer science, nanotechnology, and applied engineering, the concept of MNs was introduced in the literature report [3].

MNs are micro projections ranging 25–2500 μm in height available in different shapes with the attachment of a base for support. MNs are used to sample fluid from the body and deliver therapeutic agents to cells [4]. MNs are tiny, unique, novel, and promising devices made using microelectromechanical systems to detect, diagnose, and treat several diseases. Vaccines, nanoparticles, high or low molecular weight drugs of various categories, high molecular weight protein, and antibodies are easily loaded into MNs to deliver it into different layers of skin and deep within the skin to neutrophil Langerhans, dendritic cells for immunological effect. MNs are classified into various classes depending upon their fabrication methods, such as solid, hollow, dissolving coated and hydrogel-forming MNs. The associated advantages and limitations vary with the type of MNs desired for the targeted site of action. MNs fabrication depends on the material used and the intended application [5]. For the design of MNs, various materials have been used, such as silicon, zeolite, glass, metals, polymers and sugars. MNs array density was developed using instant microfabrication techniques (prototyping), including hot embossing, micro-molding, lithography, deep reactive ion etching, thin film deposition, etc. [6,7]. One of the most important applications of MNs in vaccine delivery to the skin is patient compliance, high immunogenic nature of skin, vaccine targeting at desired skin site, and reliable vaccine delivery methods [8]. Numerous drugs, growth hormones, insulin, vaccines, DNA, and oligonucleotides are in the preclinical and clinical stages designed as MNs [5,9].

In this present article, we review various types of MNs, different materials used for fabrication, properties of the MNs, characterization of MNs for biological and mechanical properties, toxicity assessment of MNs with various in vivo and in vitro methods, application of MNs in several diseases, as well as the regulatory aspect, marketed product, and patent of the MNs.

## 2. Types and Fabrication of MNs

**Figure 1:**
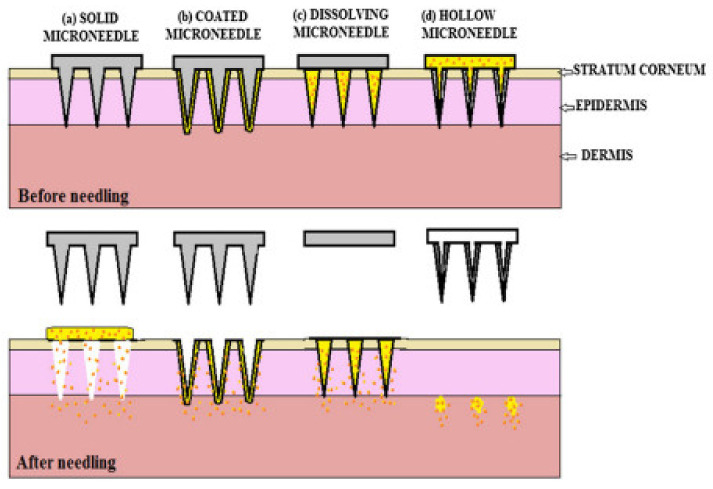
Types of MNs [14]. (Adapted from Ref. [14]).

Several types of MNs are majorly categorized based on the fabrication method and applications of MNs [10,11]. Each type of MNs has its merit and pitfalls over others for drug delivery applications. MNs are classified based on their fabrication methods [12,13]. Figure 1 and Table 1 show the types of MNs.

### 2.1. Solid MNs

The drug delivery from solid MNs is based on the “poke-and-patch” approach. The drug delivery mechanism is the disruption of the stratum corneum and the creation of microchannels by the solid MNs. After the formation of microchannels, the drug patch is applied to the skin, through which the drug is efficiently diffused through the microchannels into the skin [12].

The solid MNs were used to transfer the drug through micronized channels formed inside the skin layer and improved drug diffusion [15,16]. In solid MNs, the drug is bound to the channel, and the microchannel is closed to prevent the entry of toxic materials by using termination therapy [17,18,19]. Solid MNs serve as reservoirs for drugs [20,21]. Non-biodegradable metals can be used to fabricate solid MNs. The fabrication is conducted by forming pointed tips at the end, which helps to make micronized pores on the epidermal surface of the skin [22,23]. Materials used to prepare solid MNs are biodegradable and non-biodegradable materials such as silicon, stainless steel, titanium, nickel with polymers methyl vinyl ether polymethylmethacrylate, maleic anhydride, polycarbonate, maltose, Poly(lactide), Poly(lactide-co-glycolide), etc., [24,25]—various parameters affecting solid MN’s performance strip sharpness, insertion force, and density [26,27]. Solid MNs can be fabricated by microfabrication (micro-electromechanical system) and other methods such as using microreactors and micropumps [4,28].

### 2.2. Dissolving MNs

“Poke-and-release” is the drug delivery approach seen with dissolving MNs. In this approach, the drug is encapsulated within MNs. After insertion into the skin, these MNs are retained in the skin and not removed. The encapsulated drug is released when these MNs are degraded within the skin [26]. The dissolving MNs are not removed from the skin after the insertion as the biocompatible composition of natural, semisynthetic and synthetic biodegradable polymers such as poly (propylene), dextrin, chondroitin sulphate, polyvinylpyrrolidone (PVP) albumin, polylactic acid, poly (methyl vinyl ether-maleic anhydride) polyvinylpyrrolidone, polyglycolic acid, polylactic-co-glycolic acid, and poly (vinylpyrrolidone methacrylic acid) [4,29]. The advantage of this technology is the easy fabrication, high drug loading and convenient drug delivery.

Furthermore, these MNs do not leave any biologically harmful waste behind after dissolution, so the drug delivery is safe. The first research work on dissolving MNs and their utility were reported by Miyano et al. in 2015 as a pioneering study within this field [30,31]. The important step for the fabrication of dissolving MNs was the selection of the appropriate polymer, considering its effects on the release kinetics [32].

The literature has reported various examples regarding dissolving MNs for its synergistic drug delivery system and other techniques [32,33]. The application of the dissolving MN load cargo for delivery and improved permeation of MN array patches is clear for the vaccine delivery of influenza, adenovirus vector, etc. [34,35]. Various methods for preparing dissolving MNs include solvent casting, droplet-born air blowing, laser machining, hot embossing, microinjection molding, ultrasonic welding, and lithography [36]. The most frequently used method is the solvent casting method for the fabrication of dissolving MNs. In this method, the ultrasonic wedding fuses the polymer without heating [37]. The dissolving MNs were reported to show poor mechanical performance due to the high hygroscopicity nature [38].

### 2.3. Coated MNs

The drug delivery through the coated MNs is by a “coat-and-poke approach”. In this approach, the drug coating is applied to the MNs, and then these MNs are inserted into the skin. The drug coating present on inserted MNs gets dissolved into the skin, and after the dissolution of the drug, the MNs are removed. The advantage of this approach is that it only requires one step and has simple delivery, while a disadvantage is that a much smaller amount of the drug is delivered by this technique [39]. Coated MNs surface completely covered with the drug enables sustained release. Coated MNs were successfully studied for DNA, gene, protein, and peptide delivery [40]. These non-invasive MNs comprise steel for siRNA [41]. Important parameters that need to be optimized in these MNs preparations are the homogenous coating, stability, the method used for MN coating (spraying or dip coating) and release from the MN [15]. Gill and Prausnitz et al. showed a reduction in the surface area and a high viscosity could improve the efficiency of these MNs for drug delivery [42]. In the case of the layer coating of MNs, it has been reported that MNs are immersed in oppositely charged solutions for effective coating. The coating of antifungals on MNs was reported using piezoelectric inkjet printing [43].

**Table 1:** Types of MNs [44]. (Adapted with permission from Ref. [44]. Copyright 2019 Elsevier).

Sr. No.	Type of MNs	Material Used for Fabrication	Drug Delivery Approach	Benefits	Limitations
1.	Solid	Silicon, stainless steel, acrylic	Poke and Patch	High mechanical strength	Two-step process Poor patient compliance
2.	Coated	Stainless steel, titanium, polymer	Coat and Poke	Single step process	Limited amount of drug can be coated on to the tip and shaft of MN
3.	Dissolving/ Biodegradable	polyvinylpyrrolidone (PVP), carboxymethyl cellulose, sugar, dextran, polyvinyl alcohol (PVA), poly(lactic acid), chitosan, poly(glycolic acid), poly (lactide-co-glycolide) (PLGA)	Poke and Release	Single step process Physical removal is not required Easy fabrication process Controlled drug delivery Better patient compliance Low cost	Chances of polymer deposition in the skin with dissolving MNs. In the case of biodegradable MNs, high temperature is needed, which may affect payload
4.	Hollow	Silicon, metal, glass, ceramic and polymers	Poke and Flow	Large dose administration is possible Can be used for large molecular weight substances	Chances of needle blockage Critical fabrication process Costly
5.	Hydrogel forming	Chitosan, PVA, PLGA, poly(methyl vinyl ether-co- maleic acid)	Poke and Release	Intact removal is possible from the skin Do not leave any polymer residue Less chances of infection	Less mechanical strength Difficult to maintain the shape geometrically

### 2.4. Hydrogel Forming MNs

Hydrogel forming MNs fabricated with cross-linking polymers. The drug release approach of hydrogel-forming MNs is “poke-and-release”. The factors affecting MNs fabrication for solution parameters include a swelling index, molecular weight, and concentration of the foaming agent. This strategy was first established by Donnelly et al. for highly swellable polymers [45]. Iontophoresis, along with MN formation, enhances the efficiency of therapy [46]. The array does not contain a drug, but it imbibes through the skin layer during penetration.

This type of MNs can overcome the pitfalls of the conventional microarray technique by reducing drug loading capacity and modifying release [47]. These hydrogel-based MNs prefer sustained-release formulations [48].

### 2.5. Hollow MNs

**Figure 2:**
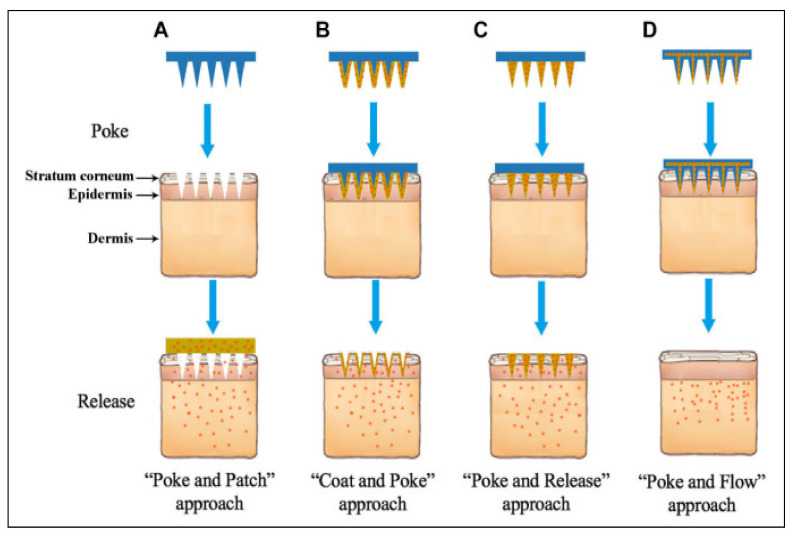
Diagrams showing various microneedle drug delivery approaches. (**A**) Solid microneedles, for skin pretreatment to create microchannels, followed by the application of transdermal patch; (**B**) coated microneedles, for deposition of drug formulations into the skin, followed by removal of microneedles; (**C**) dissolving microneedles, incorporated into the substrate of microneedles, remaining in the skin and dissolving over time to release the drugs; and (**D**) hollow microneedles, for inserted into the skin and continuous infusion of the drug through the created microchannels [56]. (Adapted from Ref. [56]).

The drug delivery approach used in Hollow MN is the “poke-and-flow”. The drug delivery from Hollow MNs is similar to the hypodermic injection. The micropump is generally used to execute them under pressure drug delivery into the skin. The advantage of hollow MN is the fast drug delivery as compared to other approaches as the drug delivery is pressure-driven. Another advantage of this technology is the painless and precisely controlled drug delivery into the skin [49]. The Hollow MNs are micron-sized hollow needles, unlike other MNs in length and diameter [50]. The usual size of the hollow MN is 30 gauge of the hypodermic needle of 300-micrometer length, and the materials mainly used in the fabrication are silicone, glass, ceramic and polymer, etc. [51]. It delivers drugs more promptly through the passive diffusion technique than the other types of MNs [52]. It is investigated that various parameters, including tip dimension, length, pressure, inner diameter, insertion and retraction of depth, affect the drug flow rate through hollow MNs [53]. Various techniques are available, such as MEMS techniques, deep reactive ion etching of silicon, deep X-ray photolithography, wet chemical etching, an integrated lithographic molding technique, and microfabrication to fabricate hollow MNs [54]. In the current era, hollow MNs are engaged in fabrication through the 3D printing method [55]. Figure 2 shows MNs drug delivery approaches.

## 3. Material Used for Fabrication of MNs

Various materials have fabricated MNs like silicon, zeolite, glass, metals, polymers, and sugars. After more continuous research, the fabrication complexity gets reduced and improves their mechanical strength, geometrical shapes, and sharpness of needles. MNs can be divided into two categories based on their fabrication material: biodegradable and non-biodegradable MNs. Table 2 shows the prototype material and its percentage used for MN fabrication.

Silicon was the first fabrication material for MNs; author Henry et al. reported the first silicon MN in 1998. The specific reason behind silicon MNs fabrication is that silicon MNs were precise, 3D structures and widely used for target drug delivery. Still, there is a high risk of breaking the needle during insertion into the skin due to its brittleness [57].

The percentage of MNs was published in various materials. Based on hits for various keywords, percentages were determined. Best Match sorted search results at https://www.ncbi.nlm.nih.gov/pubmed accessed on 30 March 2022. Some of the keywords utilized are MNs of metal glass, ceramic, silicon, and polymer [58].

### 3.1. Metal Material

The initial metal utilized in the fabrication of MN arrays was stainless steel. Metal MNs are designed by physically pushing the smallest size of stainless steel hypodermic needles through a predetermined thickness supporting material or laser cutting metal sheets into MN forms and twisting them out of the plane [58]. This type of MNs is non-biodegradable and is more suitable than silicon as it avoids brittleness. Stainless steel, palladium, titanium, and nickel are common metals used to fabricate MNs [57]. Various methods used to fabricate metal MNs are laser micromachining [59], laser ablation [60] and photochemical etching. The stainless-steel MN was prepared by author Gill et al., who drafted MNs on Auto CAD for the required shape, and the orientation of array and then laser beam ablated onto the stainless-steel sheet to cut the MN, which are manually bent at 90° from the sheet [61]. The other method, lithographic masking, was used to fabricate titanium MNs by Choi et al. They prepared a row of five in one plane MN followed by a wet etching process [62]. The comparative study was performed for the in vitro transdermal permeation of atenolol in the porcine ear skin by the silicon, stainless-steel MN array or gold—titanium MN roller; the presence of atenolol analyzed the result at the receptor site by LC-MS, and the stainless-steel MN array has a greater transcutaneous flux than the other materials [63]. The advantages of metal material are toughness and its mechanical properties within the transdermal drug delivery system.

### 3.2. Polymers

Polymers are the most promising material for MNs fabrication compared to metals and inorganic materials. The polymeric material has been used to make solid, coated and hollow MNs [64]. The polymers are less expensive, biocompatible, and have viscoelasticity to improve resistance against shear-induced breakage. The compatibility and biodegradation ability of the polymers make them one of the prominent materials of choice in the fabrication of MNs. Polymers are mostly used to prepare dissolving and hydrogel-forming MNs, whereas the poke-and-release approach is used for drug delivery. Very few polymers used as a fabricating material are seen with solid coated and hollow MNs. The dip coating, inkjet printing, and spray drying are generally used to coat drugs and polymers on MNs. In the case of dissolving needles, high drug encapsulation can be achieved due to polymer degradation-based drug release. The use of biodegradable polymers can provide sustained release due to dissolution over a period [65].

The variety of polymers used for the fabrication of MNs are polylactide-co-glycolide acid (PLGA) [66], poly-L-lactic acid (PLA) [67], polycaprolactone (PCL) [68], poly-glycolic acid (PGA) [69], hyaluronic acids (HA) [70], polyvinyl pyrrolidone (PVP) [71], polyvinyl alcohol (PVA) [72], fibroin [73], sodium alginate [74], chitosan [75], carboxymethyl cellulose (CMC) [76] and more [64].

#### 3.2.1. Biodegradable

The polymeric biodegradable MNs are made up of natural and synthetic polymers to improve drug delivery at the targeted site with a prolonged or sustainable release. The synthetic materials are mainly Polyglycolic acid (PGA), polylactic acid (PLA), polycarbonate and their copolymers (poly(lactic-co-glycolic acid) (PLGA), polycaprolactone (PCL), polystyrene (PS)), and silk, chitin, chitosan are natural biodegradable polymers used for the fabrication of MNs, which dissolves or degrades into the body by a metabolic process without producing any poisonous side effects [77]. Some other examples of natural biodegradable polysaccharides are amylopectin, dextrin, hydroxypropyl cellulose, carboxymethyl cellulose, alginate, chondroitin, and hyaluronic acid, which have been used as biodegradable MNs [58].

The fabrication of MNs using biodegradable polymers by a molding method is a novel approach that provides inexpensive and robust mass production. The polyglycolic acid (PGA), Poly-L-lactic acid (PLA) and their co-polymers polylactide-co-glycolide acid (PLGA), polycaprolactone (PCL) are used for the manufacture of biodegradable MNs as this polymer has biocompatibility, is mechanically robust, cost-effective, and resorbable. Eventually, biodegradable MNs break when poked into the skin, which has additional safety concerns as it degrades and is transported into the skin. Biodegradable polymers have minimal severe side effects. The drug can be encapsulated into MNs and then inserted into the dermal layer; as the polymer degrades or dissolves simultaneously, the drug gets released into the skin. This method does not produce any biological hazard like sharp tips waste. This polymer material can process at a low melting temperature, so the micro-molding technique is mostly used as, with these certain advantages, the polymer becomes a promising material for the fabrication of MNs. The downside with polymers is that most polymers are soft and induce catastrophic buckling during injection or blood sampling. The author Chu and Prausnitz et al. prepared the biodegradable polymer MN of an arrowhead sharp tip with the base of a metal shaft. This intellectual design fabrication requires complex processing and extra cost. Some carbohydrates are an excellent natural material resource for the fabrication of MNs as they are very effective, cheap and safe. Carbohydrates have significant biocompatibility and less toxicity and produce products with great strength. Carbohydrates are a good source of a biodegradable polymer as they show biocompatibility, are less costly and have good mechanical strength for insertion into the skin. This includes various sugars, maltose, sucrose, trehalose, mannitol, and galactose used for MN fabrication [30,78].

#### 3.2.2. Non-Biodegradable

The non-biodegradable polymers are usually synthesized using living organisms in one way or another. They have been found in the xenobiotic class, which are originally synthetic, i.e., these compounds are chemicals and do not fall into a natural polymer. Not all xenobiotics are non-biodegradable; there are many examples of biodegradable xenobiotics. We cannot say that all-natural polymers are biodegradable; lignin is the amplest natural polymer material, but it degrades by selective micro-organisms at a very slow rate. The polythioester is a biopolymer obtained by recombinant E. coli bacterial strain fermentation and can produce large quantities [79]. There are also subsequent methods available like in vitro enzymatic synthesis, i.e., immobilization of lipase enzyme of Candida Antarctica in the attendance of Epsilon-caprolactone and 11-mercaptoundecanoic acid [80]. The polythioesters are non-biodegradable as these polymers cannot produce from simple organic carbon compounds (lipids, carbohydrates) and inorganic sources (sulphur, sulphates) but from precursor substrates. In 1951 the chemical synthesis pathway of polythioester was mentioned; recent studies revealed that due to the shortage of precursor substrates and their occurrence in very few natural habitats, other than the carbon sources, the yield of polythioesters is low and not scalable for commercializing purposes. The degradation of polythioesters occurred by extracellular enzymes, that is, the enzymes located at the cell surface. The degradation of polythioesters is restricted by the molecular weight and insolubility of polymer into the water, which reduces the entry of polymer into a cell or the periplasm (cell surface); this mechanism does not cause enzymatic degradation. Polyolefin is the example of a non-degradable polymer (polyethylene) as they are found in higher molecular weights, whereas the molecular weight lowers (Hexadecane) as they are degraded by many micro-organisms [81]. Polyphenols and polyisoprenoids are water-insoluble, poor biodegradable polymers that degrade at a low rate. Lignin is a cellulose material found abundantly, and sporopollenin degrades by only white-rot fungi or other particular fungi. Polyisoprenoids include natural rubbers (cis-1,4-isoprene) obtained from the Hevea brasiliensis rubber tree and can only be degraded by Gram-positive bacteria [82,83].

#### 3.2.3. Natural Polymers

Polymers have lower tensile strength than metal and silicon materials, but have a tough nature. Many naturally occurring polymers are used to prepare MNs, including polysaccharides, proteins, and synthetic and semisynthetic polymers. These polymers are mainly used to fabricate solid, dissolvable MNs and coat the other material [58].

Carbohydrates are an excellent natural material resource for MNs fabrication as they are very effective, cheap, and safe. Carbohydrates have significant biocompatibility and less toxicity and produce products with great strength [84]. By using the master plate method, carbohydrates can be molded into suitable MNs, low in cost and biodegradable. They can be mixed with active ingredients to produce active ingredients–carbohydrates mixtures and then molded; upon insertion, the drug–carbohydrate mixture gets dissolved into the skin [85]. Many sugars can be used to fabricate MNs like maltose, sucrose, mannitol, trehalose and galactose. Maltose is mainly used to prepare MN array as it is the FDA-approved excipient in the parenteral preparation. The author, Gouhua et al., studies the in vitro study on the transdermal delivery of monoclonal antibodies using maltose MN on the human IgG protein model. The cryosection MN was pierced into the skin after the methylene blue was taken out by the maltose MN. As the MN increases in length and arrays, the delivery of human IgG increases. The other materials like starch and gelatine can also be used as they dissolve into the skin within five minutes after insertion. They can insert into porcine skin up to a depth of 200 µm, similar to the depth of rat skin. The rat model was used to investigate hypoglycemic activity by MNs and shows the equivalent hypoglycemic activity with subcutaneous injection [30,86]. The material used to fabricate protein-based MNs is collagen and its derivatives, gelatine, zein and silk, which are assumed to deliver better high molecular weight protein-based drugs and vaccines into high-capacity drug loading MNs with improved stability. These proteins are becoming a good choice for fabrication as they are mostly inexpensive and easily fabricated using micro-molding [58].

### 3.3. Natural Polysaccharides for MNs

Polysaccharides are primarily used in transdermal drug delivery due to their biocompatibility, biodegradability, cost, easy availability, ease of fabrication and sustainable delivery. They are obtained from natural sources such as plants, animals, microorganisms, etc. These include hyaluronic acid, dextran, Chitosan, and other biopolymers [87,88,89]:

(1) Hyaluronic acid MNs;
(2) Chondroitin sulphate MNs;
(3) Cellulose-based MNs;
(4) Chitin and chitosan MNs;
(5) Starch-based MNs.

#### 3.3.1. Hyaluronic Acid MNs

HA is a natural and major extracellular component of matrix and cartilage and possesses mucoadhesive properties [90]. It is widely present throughout the human body, including the dermis, synovial fluid, dental pulp, and vitreous humor in the form of non-sulfated glycosaminoglycan. It bears a negative charge and is present in water-soluble salt form. It is reported in several lengths ranging from 200, 300 and 800 µm. HA MNs are highly dissolvable in water; that serves many benefits in terms of fabrication, including high drug loading and improved economic benefits. 

Jinjin Zhu et al. studied 5-Aminolevulinic acid-loaded HA MNs for the effective pharmacodynamics therapy for the penetration of superficial tumors that showed long-term stability and deep penetration [91]. Hyaluronic acid is used as part of a combination therapy of gene and phototherapy for immunochemotherapy. The combination of p53 DNA and IR820 is readily incorporated into the HA MN patch for effective delivery [92]. Ying Hao et al. fabricated HA-based MNs to treat epidermal cancer and melanoma. MNs showed controlled release of the incorporated drug [93]. Hongyao et al. fabricated HA-based MNs to treat psoriasis and improve solubility and mechanical properties. The FDA-approved product is Microhyala, which dissolves in intestinal fluid, with degradation observed by lysosomal enzymes. Saha et al. showed the application of the HA MN array in the cosmetics and medical field [70]. A few methods used to fabricate MNs are micromolding, photopolymerization, and drawing lithography [94].

#### 3.3.2. Chondroitin Sulphate MNs

It is a natural polysaccharide compound used in the form of sodium chondroitin sulfate. It possesses certain potential features such as superior hydrophilicity and biodegradability, and hence it is used in the fabrication of dissolving MNs. It is present as the necessary component in the extracellular matrix and cartilage in the body. Fukushima et al. have developed desmopressin and rhGH-loaded sodium chondroitin sulfate and dextran MNs, which showed dose-dependent concentration [95]. In addition to this, Poirier et al. fabricated an MN array by using CS and hydroxyethyl starch. The Prepared MN array loaded hepatitis B surface antigen and QS-21 saponin as an adjuvant. A stability study suggested that at 37°, antigenicity was retained after a six month time duration, and 10% loss was observed at 50 °C [96].

#### 3.3.3. Cellulose-Based MNs

Cellulose is a natural biomaterial obtained from various sources such as wood, cotton, bacteria, and algae. It contains a beta (1,4) linkage of glucose monomers. Cellulose was reported for biomedical application. Cellulose nitrate is a film former in cosmetics [97]. The application of cellulose-based MNs has been patented in the treatment of cancer therapy [98] (US20160136407A1).

In addition, some scientists at the University of Pittsburgh and Carnegie Mellon University studied carboxymethyl cellulose (CMC) MNs for incorporating various chemotherapeutic agents and immune-stimulating agents for skin cancer [99]. These MNs have been patented for gene delivery and anti-cancer drug delivery [100]. Yong-Hun Park et al. demonstrated cellulose-based MNs fabrication by using laser writing and replica molding for transdermal drug delivery. The fabricated MNs observed a three-fold enhancement in permeability, and thus, it was considered an efficient fabrication process for even cosmetic products. Dissolvable hyaluronic acid (HA) and bacterial nanocellulose (BC) MNs have been used for dermo-cosmetic application. It was observed that an HA and BC blend provides sufficient mechanical strength to the MNs, and BC promotes the controlled release of drug molecules. The safety profile of this MN has been proven by in vivo studies [101].

#### 3.3.4. Chitin and Chitosan MNs

Chitosan is a polysaccharide prepared by deacetylation from chitin. Its linear structure of β-(1,4) linkage contains D-glucosamine and N-acetyl-D-glucosamine units. It is water-insoluble and has a molecular weight of this polymer in the range of 300 and 1000 kDa. The lower molecular weight corresponds to poor mechanical strength, and this bottleneck can be overcome by blending it with PLGA [102]. However, Chitosan shows antibacterial and wound healing properties naturally [103]. Micro-molding and electrospraying techniques were combined to fabricate these MNs to deliver doxorubicin and AuMSS nanorods. It was observed that Dox@MicroN patches showed good photothermal capacity upon increased temperature by 12 °C under near-infrared irradiation. However, these MNs have penetrated through a tumor-mimicking agarose gel and promote a layer-dependent drug release [104]. However, with the addition of the thiol group, the mechanical property has been improved, and hence thiolated MNs possess sharpness and good mechanical strength [105]. Mei-Chin Chen et al. studied bovine serum albumin-loaded chitosan-based MN for transdermal application. The prepared MN showed 95% in vitro drug release within eight days and a penetration depth of 300 μm [106].

#### 3.3.5. Starch-Based MN

Starch is a versatile biomaterial and has been explored in various applications in the biomedical field. It provides brittleness and is applicable for various topical purposes. There are numerous reports on starch-based MNs, such as that by Yujie Zhang et al., who fabricated dissolving glucose-responsive insulin-releasing MN patches for diabetes. Starch improved the mechanical strength of MNs [107]. The starch and gelatin combination used for the fabrication of MN-loaded losartan was studied as proof of concept for transdermal applications [108].

## 4. Techniques of Preparation of MNs (MNs)

Investigators have used various methods over the past few years to manufacture a wide variety of MNs. The applications of the MN are considered first while designing an MN, which includes the type of drug, its dose, desirable pharmacokinetics/pharmacodynamics, and the targets and properties of the material used for MN [109]. Achieving the uniformity and reproducibility of the needle geometry at micron-scale resolution is the main goal in the fabrication of MN to facilitate easy penetration of the needles in the skin. However, mostly as a result of the conical three-dimensional (3D) geometry and higher aspect ratio of the MNs structures, the fabrication of MNs is very challenging. The most optimized MN design and materials are considered for the fabrication of the MN. Depending on the design (various sizes and shapes), different types (solid, hollow, coated, dissolving, sharp, or flat) and different materials (silicon, metal, polymer, glass, ceramic), the manufacturing method for MNs varies [110].

**Figure 3:**
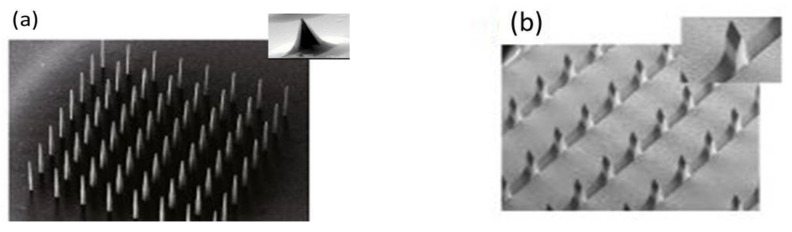
Scanning electron microscope (SEM) images of (**a**) out-of-plane MNs Source [115] (Adapted from Ref. [115]). (**b**) combined in-plane and out-of-plane MNs [116]. (Adapted with permission from Ref. [116]. Copyright 2008 Elsevier).

MNs are fabricated in various ways for different applications [6]. The two basic and primary designs of MNs are in-plane, out-of-plane, and an amalgamation of both MNs. The in-plane MNs are the simple and precisely controlled fabrication method with different lengths in the fabrication process. In the case of out-of-plane designs, the MNs are perpendicular to the fabrication surfaces [111]. Sivamani et al. are at ease generating arrays that are in-plane [112,113,114]. Figure 3 SEM images of MNs for out-of-plane and combined in-plane.

### 4.1. Microfabrication Basics

Microfabrication technology (micro-machining or micro-electromechanical) systems (MEMS) is the most encouraging method used for MNs fabrication and precise application [117]. MEMS technique exploits various tools and methods to produce smaller 3D structures with the dimensions (sub-centimeter to sub-micrometer). MN production through the MEMS technique is vastly specific and consists of multifaceted multi-step processes [118]. MEMS techniques have been potentially utilized in the biomedical fields, i.e., DNA sequencing devices, drug delivery, chemical analysis systems and biosensors [113]. A series of consecutive processes are required before the actual device is generated in the MEMS process. The three primary techniques in the MEMS technology include: (i) thin film of material deposition on a substrate; (ii) application of the patterned mask on the top of a film by photolithographic imaging; and (iii) selective etching of the films for masking [119,120].

### 4.2. Thin Film Deposition

Thin-film deposition leads to the addition of the material in the thin-film layer over the substrates. These thin layers could act as spacers with a few nm thicknesses to 700 µm [121]. Thin-film deposition occurs in two types, subject to whether the process is mainly chemical vapor deposition or physical vapor deposition (PVD) [122]. In the PVD process, the raw materials, i.e., solid, liquid or vapor, are freed and transferred directly from source to material to be coated and the substrate through the gas phase, e.g., thermal evaporation, ion plating and sputtering. In thermal evaporation, the source (e.g., aluminum) is heated by a radiofrequency or electron beam and the silicon wafer is located inside a vacuum chamber. A source boils on heating, and vapors are condensed on the substrate surface to form a film. In the sputtering technique, high-energy particle bombardment atoms or molecules are expelled from the target material. The expelled atoms or molecules could condense on the substrate, such as a thin film [123]. In ion plating, the material used for coating is ionized and vaporized with the assistance of an electric arc and then forced toward the target at high speed [124]. A thin film is formed in the CVD process by a thermally induced chemically driven reaction among the inert-carrier gases in a chamber and hot substrate. This flexible method works at atmospheric pressure at moderately lower temperatures [125,126]. The two most common CVD technologies in MEMS are the LPCVD (Low-pressure CVD) and PECVD (Plasma-enhanced CVD). The LPCVD could allow uniform deposition of many thin-film materials on substrates without damaging effects on film homogeneity at higher temperatures, >600 °C. Conversely, PECVD functions at low temperatures (200 to 400 °C) due to thermal cycle concerns or material limitations, but the films grow faster [127]. In conclusion, a specific deposition process depends on several factors, e.g., source, substrate structure, apparatus, working temperature, deposition rate, and production time. Afterward, the thin layer deposition is decorated using photolithographic techniques and then etched away to create the final structure [119,120].

### 4.3. Photolithography

**Figure 4:**
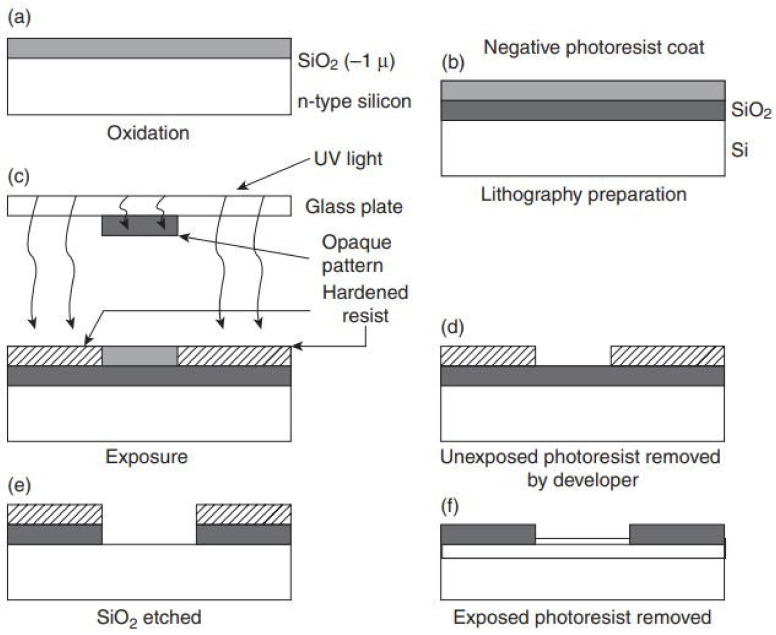
Sequential processes in transferring a pattern/design onto the substrate surface [114]. (Adapted with permission from Ref. [114]. Copyright 2013 Elsevier).

**Figure 5:**
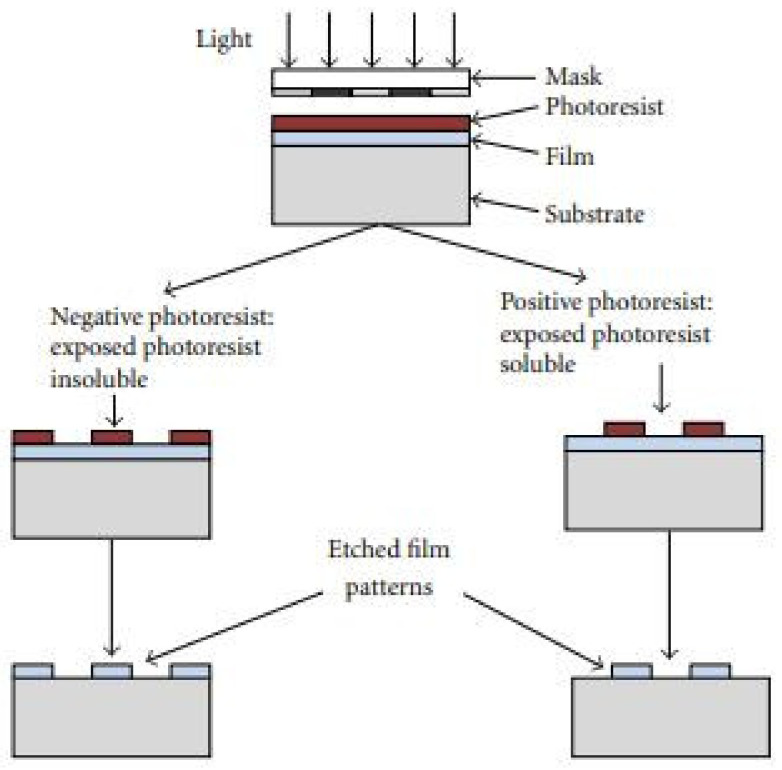
Positive and negative photoresist [133]. (Adapted from from Ref. [133]).

Most of the developments in microelectronics and micromachining fabrication start with lithography. The different types of lithography include ion beam lithography, photolithography, X-ray lithography and electron beam lithography. The maximum widely utilized type of lithography is photolithography [128]. Photolithography is used to decoratively create dissolving, hydrogel, solid, and hollow MNs. This method is also used to manufacture silicon MNs and polymer MNs by making an inverse mold based on MNs structure [14]. This technique transfers copies of a master design on the substrate surface of some material, e.g., a silicon wafer. For this purpose, a thin layer of oxide is developed onto the surface of the silicon wafer by heating it at 9000–11,500 °C in the occurrence of steam or humidified oxygen steam. This is followed by the deposition of a thin layer of a photoresist organic polymer, sensitive to ultraviolet radiation on the silicon wafer’s oxide surface (Figure 4a). A spin coating process carries out this deposition (spun, 1500 and 8000 rpm) to produce a photoresist of a well-defined thickness (Figure 4a) [129]. The solvent in the resist layer is taken out through heating at 750 and 100.80 °C for 10 min after the spin coating step. Along with solvent evaporation, this process indorses devotion of the photoresist layer to the silicon wafer. When the solvent is taken out, a glass plate (transparent) coated chromium pattern (opaque) and a photomask are positioned to contact the photoresist-coated surface [130]. The silicon wafer is exposed to UV radiation (150 and 500 nm), shifting the photomask’s design to the photoresist-coated wafers (Figure 4c). The radiation treatment stimulates a chemical reaction in exposed sections of the photoresist, which are of two types; negative and positive (Figure 5). The solubility of the exposed photoresist is altered after this reaction. Afterward, in the course of the development processes, this resistant region could be dissolved by a rinsing solution that removes either the exposed areas or the unexposed areas of the photoresist, either by wet (solvent) or dry (plasma or plasma or dry vapor phase) techniques [131]. Thus, it leaves a design of photoresist-coated and straightforward oxides on the wafer surface (Figure 4d). Subsequently, unwanted photoresist left after the advancement process is detached by the de-scumming-induced oxygen-plasma treatment called de-scumming [110]. The final oxide design (positive or negative) is then a photomask pattern used as a mask in subsequent processing steps (Figure 4f) [132]. In the MEMS method, the oxide is utilized as successive masking to form either a new layer on which further layers are to be built, or further etching to form deep 3D holes, subsequent in a complete 3D structure or device [120].

### 4.4. Etching

Etching is an important process to fabricate the final and optimized functional type of MEMS structure onto the substrate after lithography. This technique creates a design on a substrate surface by incising the thin films (unprotected parts) previously deposited/placed on a substrate and/or removing layers of the substrate itself by using strong acid or a physical process. It is mainly carried out after the photolithography process, through which a uniform layer of photoresist is deposited over the substrate and patterned for fabricating the etching pattern [4]. Any photoresist layer or a film resistant to the etching process can be used as the masking material, e.g., silicon dioxide, silicon nitride, and metal films. Etching is also used to determine the tapered shape of MNs tip, the size of MNs base and gap among the MNs before the etching process and the length and shape of the MN after the etching process [134]. Multiple applications use etching, including silicon micromachining [135], fabrication through nanoscale etching [136], IC fabrication [137], biosensors [138], accelerometers [139], phosphoric acid etching of the human enamel for enhancing resin adhesion for dental applications [140], PDMS [141] and the development of microfluidic devices via the etching of Parylene [142,143]. The critical parameters of an etching process are structuring with high resolution, cutting direction of the etchant and higher aspect ratios. In general, the etching processes are dry etching and wet etching, subject to the physical state of the etchant [4].

#### 4.4.1. Wet Etching

**Figure 6:**
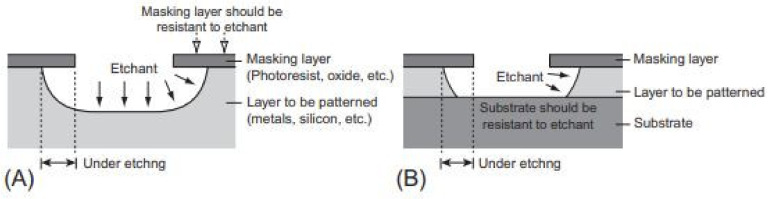
Wet etching (side view). (**A**) indicates a case where a substrate or a thick material is being etched, and (**B**) shows a case where a thin film deposited on a substrate is patterned [134]. (Adapted with permission from Ref. [134]. Copyright 2014 Elsevier).

**Figure 7:**
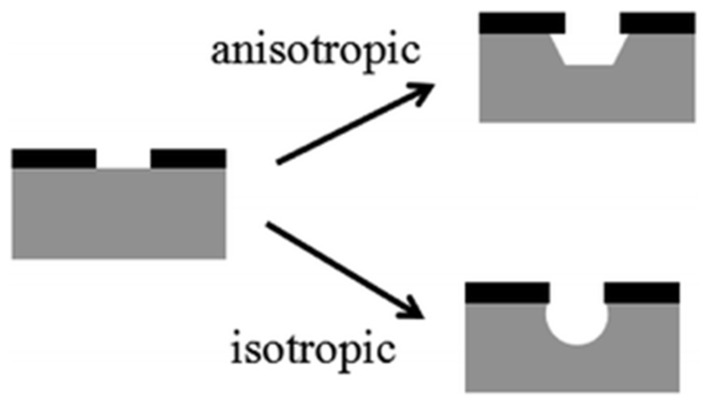
Etching profiles generated with [146] isotropic and anisotropic etching. (Adapted from Ref. [146]).

Wet etching is a patterning method. The material (typically a silicon wafer) or metal is removed/etched by submerging in a liquid bath containing a chemical solution or etchant [144]. In this process, the film designed/patterned by etching is covered by another layer of pattern that is resistant to an etchant (Figure 6A,B). The layer to be patterned is the metal film, and the masking layer is often a designed/patterned photoresist film [145]. The etchants used for wet etching are classified into isotropic and anisotropic (Figure 7).

**Figure 8:**
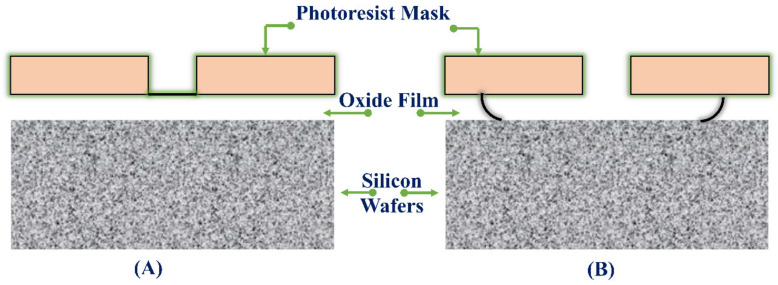
(**A**) Oxide film onto the silicon wafer with established photoresist mask, (**B**) after prolonged wet etching; the etch has developed under the mask (not to scale) [120]. (Reprinted from Ref. [120]).

The isotropic etchants engrave the material, such as oxide, aluminum, nitride, polysilicon gold and silicon, at the same rate in all directions. Hence, they take off material horizontally under the etch mask at the same rate as they etch through it. For instance, a thin oxide film on a silicon wafer is etched with isotropic etchants (hydrofluoric acid) that etch the oxide quicker than the underlying silicon, as shown in Figure 8. In amalgamating with water or methanol, isotropic etchants such as hydrofluoric acid, phosphoric acid, and nitric acid could be used [147].

**Figure 9:**
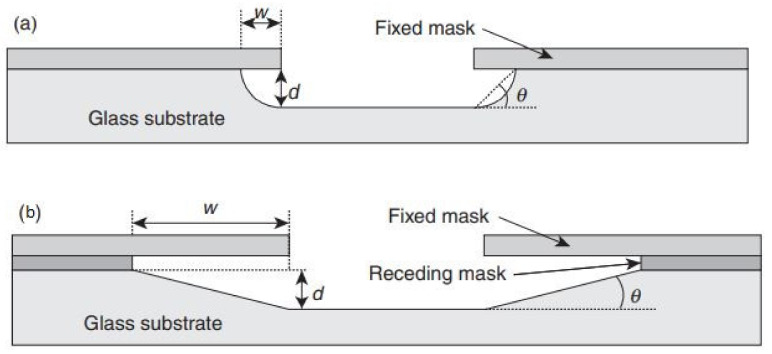
Schematic illustration of etching of glass by (**a**) Si passive etch mask, and (**b**) Schematic representation of etching of glass with a bilayer mask made of a fixed mask and a receding mask [148]. (Adapted with permission from Ref. [148]. Copyright 2013 Elsevier).

Rounded side wall microchannels are produced by isotropic wet chemical etching. The application of titanium as a receding mask through the wet etching process leads to adjusting the shape and angle of the sidewall of the microchannels (Figure 9). The etch rate and etch duration control the channel’s depth, while mask opening to twice the channel depth estimates the width of the channel. A thick layer of negative photoresists (i.e., SU-8) may be a simple, low cost and suitable masking material in case of some shallow etches.

During the wet etching isotropic process, if the etching time is prolonged or when the etchant is the strong base solution, the photoresist could not offer enough protection and easily get damaged. For example, the photoresist layers are effortlessly etched in a wet etching of silicon where potassium hydroxide (KOH) or tetramethylammonium hydroxide (TMAH) is utilized. Hence, they have not functioned as the masking layer. In such a case, a multistep etching process is employed where the silicon dioxide is designed through a photoresist as the masking layer and HF as the etchant. As a result, silicon dioxide is the vastly resistant mask to KOH or TMAH, and the silicon layer is then etched [149].

HF is employed as the main etchant for all types of silicate glass. The chemical reaction for etching is shown below:

SiO_2_ + 6HF → H_2_SiF_6_ + 2H_2_O

It is usually used to etch the oxides, and in strong concentrations, it speedily strips oxides [150]. Buffering with ammonium fluoride is known as buffered HF (BHF), or buffered oxide etch (BOE), which is used to pattern oxides. As a result of the buffering of HF, its etch rate is more precise and controlled, and it could not peel photoresist as it is more concentrated than HF [151]. In mixtures with other compounds, phosphoric acid is utilized to etch either nitride or aluminum substrate, where oxide is used as the mask [152]. Gold is typically etched with an iodine-based solution [153]. Noble metals are etched by aqua regia, a mixture of hydrochloric and nitric acids (3:1) [154].

Anisotropic etchants attack the material at diverse rates in different directions, so they are faster in a favored direction at producing more controlled shapes [121,155]. The most common anisotropic etchants are potassium hydroxide (KOH), tetramethylammonium hydroxide (TMAH), ethylenediamine (EDP) and hydrazine [156]. The crystal orientation of the substrate or wafer decides the structures formed in the substrate.

Depending upon the exposure of the crystal faces of a few crystalline materials, they remain etched at diverse rates. Silicon is a single-crystal material having different crystal planes that allows very high anisotropic etching at different rates. KOH or TMAH is regularly used as an anisotropic etchant. Using KOH, the anisotropic etching of silicon wafers results in the most common crystal orientation (100). The etch rate of a 30% KOH solution in ˂100> directions is 0.8 μm/min, which is >150 times higher than the ˂111> directions [157], while the (100)/(111) etch ratio of TMAH is approximately [158] 10–50. Oxide and nitride both etch gradually in KOH. Silicon dioxide could be employed as an etch mask for short periods to form narrow grooves and pits; however, KOH etches silicon dioxide a few nm/min, which is considerable for some applications. For longer periods and deeper etching (>100 μm), silicon nitrides are an enhanced etch mask as it etches more gradually in the KOH. TMAH has good etching discernment between silicon dioxide and silicon [159]. On the requirement of a deep etching with a thickness of a silicon wafer (500 μm), a thermally developed oxide layer could be used as a mask.

**Figure 10:**
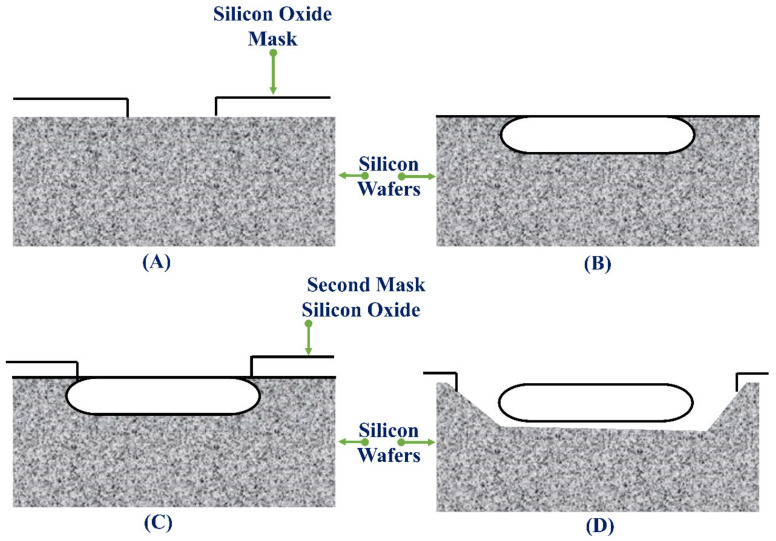
Concentration-dependent etching methodology: (**A**) mask for the boron diffusion, (**B**) oxide mask stripped succeeding diffusion, (**C**) mask for the KOH etching, (**D**) boron-doped structure expelled by the KOH etching [120]. (Reprinted from Ref. [120]).

The amount of boron in silicon affects its rate of etching. An increased amount of boron in silicon reduces the etching rate in KOH by a greater extent and further stops the etching of the boron-rich silicon, called as concentration-dependent etching method [160]. The diffusion method is used to introduce the boron impurities in silicon. A silicon oxide mask is introduced on the surface of the silicon wafer. It is designed such that the surface of the silicon wafer is exposed for the introduction of boron (Figure 10A). The silicon wafer is now positioned in a furnace in connection with a boron-diffusion source; nevertheless, it is required to retain the time in the furnace as small as possible. The boron atoms transfer to the silicon wafer for 15 to 20 h. After the boron diffusion is finished, the oxide mask is stripped off (Figure 10B). A second mask is deposited and designed over the silicon wafer before immersing it in a KOH etch bath (Figure 10C). The KOH etches around the boron-doped silicon and the silicon, which is not sheltered by the mask (Figure 10D).

**Figure 11:**
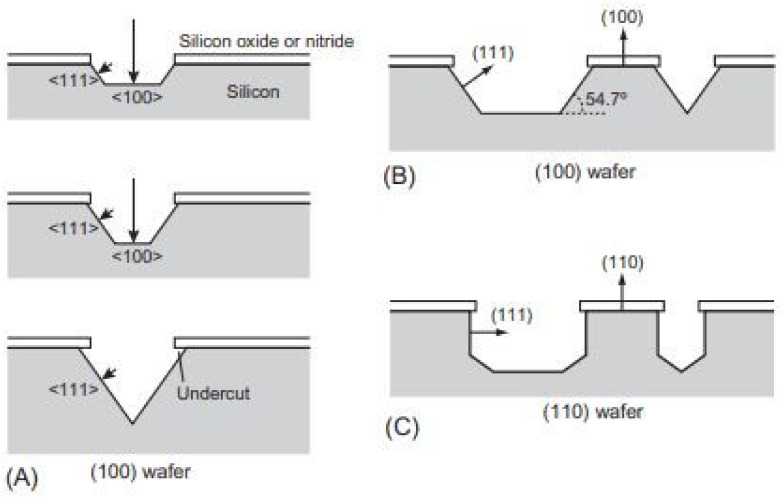
The anisotropic silicon wet etching (side view). (**A**) Etching in the ˂100> direction is much faster than in the ˂111>direction. (**B**) An angle of 54.7° is observed between (100) and (111) surfaces. (**C**) Vertical walls are created with (110) wafers [134]. (Reprinted with permission from Ref. [134]. Copyright 2014 Elsevier).

A typical anisotropic etching process with a (100) silicon wafer is shown in Figure 11 A. Anisotropic etching on a silicon wafer continues in the ˂100> direction and is directed by the (111) surface on all four sides. The (100) surface will shrink as the four (111) surfaces meet at the apex of the inverse pyramid structure during the etching process. Etching in ˂111> direction is remarkably slower and is detected as an undercut. The shape of the etched cavity could be roughly assessed by considering the angle between a (100) surface and a (111) surface, which is 54.7° (Figure 11B). The etching of a (110) wafer is further complex than that of a (100) wafer (Figure 11C). Several studies have been carried out to examine overall cases of anisotropic silicon etching [161,162,163]. Good predictions for shapes of etched wafers can be found using numerical analysis [164,165,166].

Wet etching is used to produce silicon MN, metallic MN and hollow MN arrays of a sharp tip [155]. However, the crystal planes in silicon limit anisotropic wet etching [167]. The etching rate in wet etching is considerably faster than in dry etching.

#### 4.4.2. Dry Etching

Dry etching, or plasma etching, is the etching process in which an accelerating motion of an ion species to the substrate combined with a masking process is used to physically or chemically etch the target materials. This form of etching is carried out at little pressure using inert or reactive gases. The most commonly used dry etchants are hydrogen fluoride [168], fluorocarbons [169], xenon difluoride [170], oxygen [171] and boron trichloride [172]. Dry etching is considered a huge scale integration (VLSI) process as it could be more exactly controlled by regulating parameters like gas pressure, temperature and electric field distribution. Dry etching is a method classified into two types: reactive ion etching (RIE), which involves chemical processes and ion-beam milling, which involves purely physical processes [25]. Depending on the pressure in the plasma chamber and the electric field that provides direction to the ionic species in the plasma, this process can be either isotropic or anisotropic [173]. Dry etching methods were used for patterning metals such as Aluminum [174], Copper [175] and Titanium [176], organic materials [177], Polymers such as PDMS [178], Parylene [179], PMMA [180], Polycarbonate [181], Polyimide, [182] and SU-8 [183], silicon and silicon dioxide [184], silicon nitride [185] and, Glass [186].

#### 4.4.3. Reactive Ion Etching (RIE)

In RIE, a plasma generates high-energy ions in a chamber that reacts chemically with the substrate to be etched. RIE is a form of isotropic etching, primarily embodied as barrel etching; conversely, it is often utilized as an anisotropic etch. In this form, the reactive ions are accelerated toward the substrate to be etched [132]. The electric field accelerates ions, and etching is enhanced in the direction of the travel of the high-energy ions. The degree of etching in an RIE system depends strongly on reactive gases, gas flow, pressures, temperatures, RF power and DC bias [187]. The above parameters have been thoroughly studied to fabricate such deeper structures. Using the RIE system, various materials such as silicon, oxide, and nitride can be etched to form pits (up to several microns) of arbitrary shape, deep trenches, and holes with vertical walls [188]. Despite the anisotropic wet etching, RIE is made inadequate by the crystal planes in the silicon. The table shows different etchant gases used for the plasma etching of various films. Various chemicals are used in plasma etching which ultimately results in etchant gas like Chlorine, Oxygen, Fluorine etc. [189].

The etching rate in RIE is low; hence, to achieve a high width-to-height ratio, a deep RIE (DRIE) process was introduced. A deep RIE (DRIE), often called the Bosch process, was introduced to generate high aspect ratios, height-to-width ratios, and structures in amalgamation with chemical vapor deposition (film forming process). Fabrication parameters can be optimized using the BOSCH process to achieve a high aspect ratio; high etch rate, straight sidewalls and small sidewall scalloping. The possibility of getting non-vertical, tapered sidewalls is limited [190].

In order to generate high aspect ratio troughs or beams with vertical walls in a substrate, two different phases that work repetitively at a reasonable frequency are employed [191]: (1) Etching: employing an isotropic plasma-enhanced etching method, the substrate is etched; (2) Deposition: In the second phase, the entire substrate is deposited with an inert protection layer (e.g., C_4_H_8_) to preserve the etching of the side walls when the plasma etching phase is repeated for the next cycle of fabrication. The Bosch process typically creates undulating sidewalls due to the above two steps. The Bosch method is appropriate for the manufacture of off-plane MNs. This method is also used to yield hollow MNs with a lumen of several hundred µm widths to height ratio of 30:1 [192]. Although wet etching can decrease fabrication costs more than dry etching, distinct and sharp MN tips are fabricated by uniting isotropic dry and anisotropic wet etching [49].

**Figure 12:**
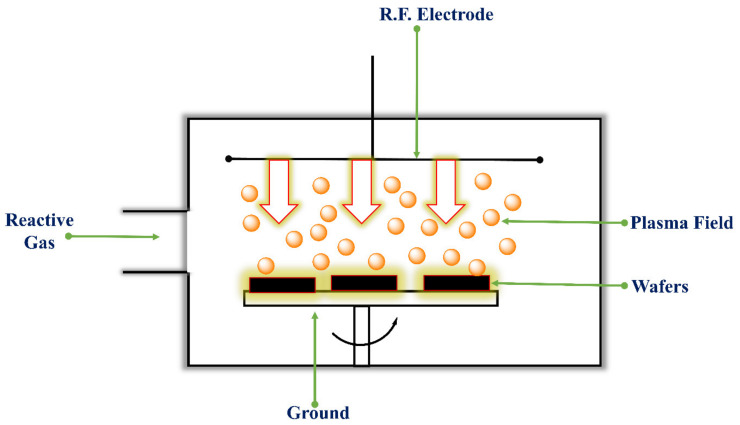
Planar plasma etches configuration method. The wafers are held on a grounded chuck close to the RF electrodes. Reactive gas introduced in the chamber is ionized, and the ions help in material removal [189]. (Reprinted from Ref. [189]).

Research by Henry et al., in 1998, fabricated solid silicon out-of-plane MNs arrays employing the DRIE process. The chromium dots formed the masks on the silicon wafers. The patterned silicon wafers were etched using a reactive ion etcher using SF_6_ and O_2_ gas as an etchant at a pressure of 150 mTorr and power of 150 W for a run time of about 250 min. These parameters resulted in deep vertical and lateral etching forming MNs from the regions protected by the chromium masks [193]. Howells et al., 2022 fabricated solid silicon MN and hollow silicon in-plane MN arrays with a 54.7° sidewall etch angle from a simple single wet etch process. MNs were fabricated using double-side polished, boron-doped, 300 µm thick (100) orientation silicon wafers with a thermal silicon dioxide layer on both front and back sides. Photolithography processes were used to pattern devices onto silicon dioxide. Using ICP, the device pattern was etched into the silicon dioxide hard mask. The wafer was flipped, and again, the device was patterned and etched. The whole wafer was submerged in 44% KOH solution for etching for 5 h and then removed. V-shaped grooves were achieved as the KOH concurrently etched together the front and back sides of the wafer, which further intersected to form a sharp pyramidal six-sided MN tip. Via bonding of two grooved MNs together, a hollow MN was designed using ICP. The MNs arrays established efficiently pierce in the skin, lacking significant indentation, thus enabling actual delivery of drugs using solid MNs or direct injection using hollow MNs. Effective insulin and hyaluronic acid delivery into the skin was achieved using these MN arrays [194]. Figure 12 shows the Planar plasma etch configuration method.

### 4.5. Ion-Beam Milling (IBM)

**Figure 13:**
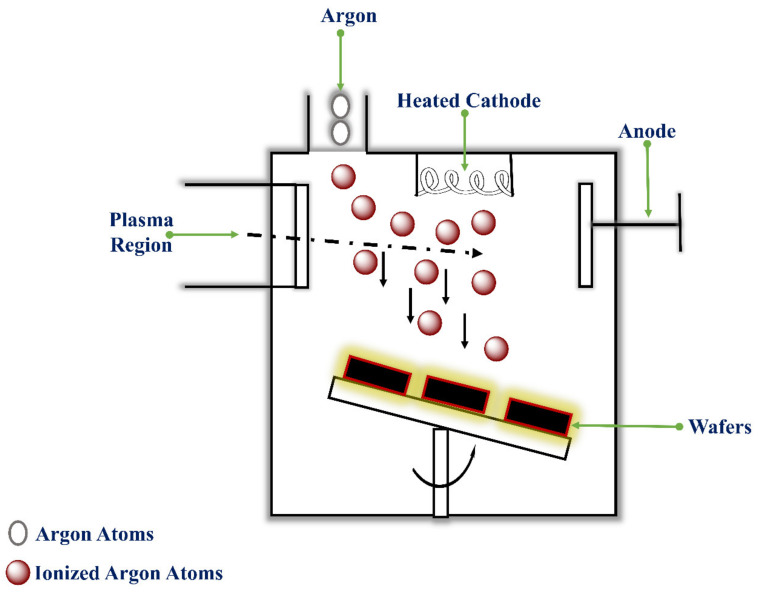
Representation of the ion beam etching process methods. Ar gas introduced in the vacuum chamber was ionized by bombarding with electrons. These ions are then directed onto the wafer, where they eliminate material by physical bombardment [189]. (Reprinted from Ref. [189]).

The Ion-Beam Milling method, in which inert ions are accelerated from a source to physically eliminate the material etched from the wafer. They are of two types; showered-ion-beam milling (SIBM), and concentrated ion-beam milling (FIBM) [195]. In SIBM, energetic ions are poured over the whole substrate. In FIBM, ions are concentrated on a spot directed to a particular workpiece part [196]. Although SIBM is slower and more controlled, it can be used as RIE. In ion-beam milling, inert gas ions, usually Ar, are used as they exhibit higher sputtering yields due to heavy ions and avoid chemical reactions, e.g., Ar, O_2_, N_2_, Xe [197]. The process is not selective or specific, but it is highly directional. Figure 13 shows a Representation of the ion beam etching process methods.

## 5. Characterization of MNs

The characteristic feature of MN advancement is its diversified application. The basic characteristic includes dimensions, i.e., size, shape and geometry. The material (metal, ceramic, silicone) used in designing MNs also has a significant role in the characterization of MNs [198].

These characteristics include: (1) Deep penetration of intact MN into the skin tissue. (2) Optimized dimensions, as the short needle would not be completely pierced into the skin tissue, and with a long needle size, there might be a risk of breaking (due to lack of strength and rigidness) before insertion into the skin [199,200].

### 5.1. Morphology and Geometry

Morphological characteristics like shape, length, base diameter, and a tip diameter of MN are observed.

#### Scanning Electron Microscopy (SEM)

The study of geometry and dimensions was conducted on two types of MN, i.e., maltose MN and DermaRollerTM (metallic), on a Scanning electron microscope (Hitachi S-4100) for MN imaging. These MNs samples were directly imaged without coating with DermaRollerTM, and detachment of the head embedded with MN from a needle holder was executed, and samples were placed in SEM with 15 eV accelerated voltage. The measurement of the dimensions was recorded by software—Vantage 1.3 (Noran Systems, Middleton, WI, USA). The observed results were as follows; maltose MN consisted of pyramidal shape, 497.41 ± 31.10 μm length, 197.60 ± 17.53 μm base diameter and ~8 μm tip diameter, whereas DermaRollerTM MN had conical shape, 699.38 ± 70.72 μm length, 127.88 ± 15.96 μm base diameter, ~15 μm tip diameter [201].

Amer RI et al. characterized polymeric MNs of Sodium alginate by conducting an SEM analysis. The geometry of the MN was confirmed from the master mold where the visualization of MN length is 600–650 μm, the base width of 300–350 μm with 100–125 μm interspacing. Deformation was observed slightly, i.e., needles were more wide and flat; this was associated with sampling preparation with a low level of electron beam and magnification, which resulted in the interaction of both the sample and electron beam, causing sample degradation by melting.

Uniform sharp tips inter-spacing dimensions of MN were observed and confirmed by a photograph of a light microscope (MN cross-section sample) [202].

### 5.2. Mechanical Integrity

#### Thermogravimetric Analysis

A TGA was performed for sorbitol, polyvinyl alcohol, and MN patch to ensure the thermal stability of components of MNs where a 3% loss in mass, caused by the evaporation of adsorbed water, was observed for a powdered sample of polyvinyl alcohol (PVA) with temperatures ranging from 25 °C to 200 °C. More than 60% mass loss in PVA was recorded at 300–370 °C, indicating thermal degradation of PVA, and an additional 30% loss in mass at 370–470 °C occurred due to the decomposition mechanism. Sorbitol under thermal exposure reported mass loss <1% at 230 °C and represents thermal stability. A further 50% reduction in mass was observed with a temperature rise of 230–350 °C, representing thermal degradation. The MN patch showed the approximate 85% mass loss is similar to PVA as it consists of PVA in large amounts. About 6% mass reduction at 65–240 °C occurred due to water removal and melting. At 300–370 °C, organic combustion caused mas sample reduction of ~48%. At 380–480 °C the sample showed second phase degradation. This study concluded the efficient thermal stability of sorbitol, polyvinyl alcohol, and MN patch [203].

### 5.3. Swelling Property

The swelling property signifies the mechanical integrity of the MN. The swelling study is carried out as follows; where a blank and dry patch was taken from a known mass (Wd), and was immersed in a petri dish containing phosphate buffer saline solution with pH 6.8. The immersion continued until the swelling caused disruption to the structure and thus the structural integrity collapsed due to water uptake (approx. 30 min). The patch was taken out of the petri dish, and using filter paper, surface water removal was conducted [204]. The swollen patch was weighed (*W_s_*), and the determination of the percentage of swelling was calculated using the following formula:

$$W\%=\frac{\left(W_{S}-W_{d}\right)}{W_{d}}\times \left(100\right)$$

**Figure 14:**
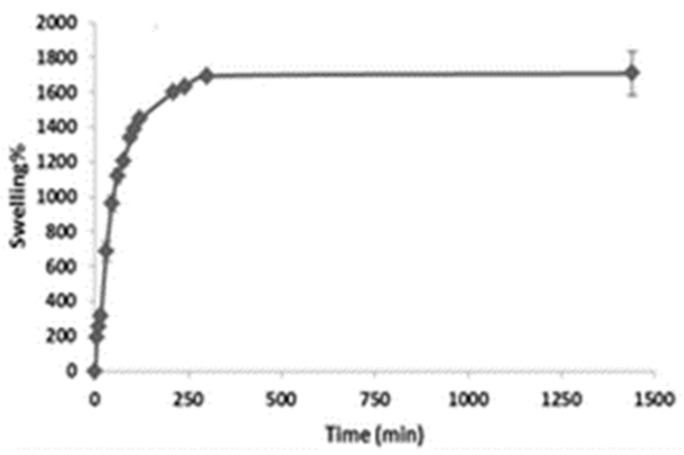
Swelling curve for crosslinked hydrogel films prepared from aqueous blends containing 20% *w*/*w* PMVE/MA, 7.5% *w*/*w* PEG and 3% Na_2_CO_3_ based on the increasing mass of the swelling array expressed as a percentage of the mass of a dry array (Means ± SD, n = 3). Adapted from ref [45].

The super swelling MN arrays fabricated using an aqueous mixture containing 20% *w*/*w* Gantrez S-97, 7.5% *w*/*w* PEG 10,000 and 3% *w*/*w* Na_2_CO_3_ were subjected to swelling studies PBS pH 7.4 buffer. First, the weight of the MN was measured in a dry state at a zero time point and then immersed in PBS pH 7.4. The MN films were removed at specific time intervals, surface fluid was wiped, and the MN film’s swollen mass was measured. The results showed the highest swelling and fast achievement of the equilibrium state by the super swelling MN compared to the control formulation (15% Gantrez AN-139, 7.5% PEG). At equilibrium, the super swelling percentage of the super swelling MN compared to the control MN was 1708% and 1071%, respectively. Thus, the above study proves the mechanical integrity of the hydrogel MN array when administered transdermally [45]. Swelling of the polymer network hydrogel is presented in Figure 14.

### 5.4. Drug Release and Drug Distribution

MN design aims to ensure drug delivery into the epidermis and dermis of the skin by piercing the stratum corneum. The skin staining technique uses methylene blue stain to confirm these MN holes by piercing the MN. Preparation of methylene blue solution of 10 mg/mL by adding methylene blue powder(M9140, Sigma-Aldrich, St. Louis, MI, USA) and distilled water. For biological stains, methylene blue dye is used. It acts as it binds with the protein in the tissue; due to its hydrophilic nature, it is not absorbed by the stratum corneum hydrophobic nature [205,206].

When coated, PLA MN and dissolvable MN were removed from a skin sample, the deposition of methylene blue on the skin sample was conducted for 10 min on the skin surface, and leftover methylene blue solution was wiped out with ethanol (24,194, Sigma-Aldrich), leaving dye stains in the punctured region of the stratum corneum and highlighting them [67].

MN drug delivery aided the rapid dissolution of the drug into the body. Drug distribution enhances the efficiency of drug release. The interaction of polymer and drug molecules was carried out by multiscale simulation influencing MN drug distribution. The study was carried out with sulforhodamine B (SRB) as a model drug, along with Hyaluronic acid (HA) and polyvinyl alcohol (PVA) for fabricating MNs. Dissolvable MNs were produced to study controlled drug distribution in those MN patches. These observations were studied using an optical microscope under an objective lens by viewing the side and top angle of the MN array. The analysis of SRB in both MN patches was studied and observations made were as follows; limited drug distribution in PVA, as drug molecules, are seen concentrated at the tip, and mild concentration was seen in the middle portion, with no drug molecules at the base of the needle. Conversely, Hyaluronic acid showed poor drug diffusion control ability. Here, SRB distributes largely into the needle body and bottom plate; it has a poor concentration at the needle tip; owing to the high water solubility of HA with a high compatibility drug molecule, Hyaluronic acid faced a significant challenge in achieving controlled control drug distribution [207].

## 6. Mechanical Properties of MNs

Mechanical strength is an important aspect for MNs to perform their function. Mechanical property is often determined by compression strength [208]. The compression strength of MNs assesses the mechanical property of MNs. The measurement of the robust nature of MNs is their integrity for skin penetration. Here, metal-coated MN is assessed by Yang SJ., et al. for compression strength. The MNs skin penetration was performed using porcine skin. The texture analyzer was used with a trigger force of 100 N and 12 mm working distance (from the probe to the skin) for the MNs skin patch (MNP) testing. The probe holding this patch descended at 100 mm/min speed until it reached 10 mm from the target, slowing down towards 10 mm/min speed with 2 mm from the surface. As it approached the skin surface, the needle points penetrated 700 μm. When full penetration of the MNs patch with porcine skin took place, the detachment of the MNs patch from porcine skin and the images were reviewed.

The calculation of compressive strength was made when the MNs patch was penetrated to a length of 50% into porcine skin. There was a slight MNs patch stress change by a 43% compression rate. An optical microscope represented MNs patch deformation. Flat PVP- hydrogel has a compression strength of 268 kPa, while PVP-MNP has 284 kPa of compression strength; PVP-MNP’s higher compression strength is due to the larger force applied per unit of arrangement. The compressive strength of Ag coated PVP MNs 278 kPa, and Au coated PVP MNs 276 kPa are slightly less than PVP-MNP due to polymeric chain degradation with polymeric chain heat created during the process of degradation. These Ag and Au coated PVP MNs have higher strength than flat PVP hydrogel. The MNPs have perfectly penetrated the porcine skin, seldom broke or deformed. Furthermore, complete skin penetration occurred at the surface without breaking at contact. The slight reduction in length < 1% when penetrated to 700 μm was observed before (~701–703 μm) and after (~692–695 μm) MNs length skin penetration. The shape was retained well (slight deformation). Results represented effectiveness in drug delivery as PVP-based MNPs are prepared by radiation and have MNs of sufficient strength and flexibility required for insertion through the stratum corneum and to reach the dermis [200].

## 7. In Vitro and In Vivo Evaluations of MNs

### 7.1. In Vitro Studies

In vitro studies for an assessment of the MNs include penetration and permeability studies. The skin distribution technique evaluates the uniformity and depth of penetration by MNs. The patch is placed on the hairless skin of a Rat or Pig and immediately removed. The skin site is dyed with a cotton swab soaked with India ink. This skin is then stored at −20 °C on dry ice for three days up to processing. The skin is cut in a section of uniform size (6 mm, with a cryotome instrument). The number of stained pathways in each section is counted. The percentage of MNs to have penetrated the skin can be plotted as a function of depth, and the depth at which 50% of the MNs penetrated can be extrapolated [209]. Permeability studies of MNs can help to predict how MNs can help improve drug delivery. In vitro methods for a permeation study are usually carried out by diffusion cell apparatus and rat skin. Kocchar et al. [210] conducted an in vitro permeation study of Bovine Serum Albumin (BSA) MNs on abdominal rat skin with the help of a water-jacketed horizontal diffusion cell. Overnight hydrated skin in 0.005% phosphate-buffered saline was placed as stretched on 10 layers of Kim wipes to absorb and provide mechanical support like tissue [211]. MNs, having different concentrations, were applied to a taken rat skin as a sample, and MNs without any BSA was used as a blank. BSA solution in propylene glycol (PG) was used as a standard to compare the release of BSA by passive diffusion and MNs. The MNs were fixed on the skin with the help of scotch tape. This skin was placed in the middle of the two compartments. About 4.5 mL of receptor solution was placed in the receptor compartment and stirred continuously at 250 rpm. Then, 1 mL of receptor solution was withdrawn at each sampling point. The removed release samples were centrifuged at 10,000 rpm for 5 min. UV A215-A225 method164 calculated the permeation of BSA through the skin. Another study checked Doxorubicin MNs’ permeation on the mouse skin spread on a glass slide. The MNs were removed, and a confocal scanning microscope observed the skin to check the penetration of drugs into the skin [212]. In the case of dip-coated MNs, the content of coated material (Lidocaine) was determined by HPLC. The coating of MNs was desorbed into a diluent, and the resulting solution was injected into HPLC, and freebase Lidocaine was used as the standard for the quantification [213]. The study carried out by Kumar et al. [214] used MNs for piercing the skin and permeation of nanoparticles through the skin. The dorsal skin on BALB/c mice was made free of fatty layers. MNs roller was then perpendicularly rolled in four lines for five times, twenty times each. The applying pressure was constantly monitored and kept between 350–400 g. This treated skin was clamped between the donor and receiver compartment of the Franz-diffusion cell. The donor compartment was filled with the pCMV-β-coated nanoparticles in water. The receiver compartment was filled with 5 mL of PBS (pH 7.4). The temperature of the setup was maintained at 37 °C with a continuous circulation of water. At several time intervals, 150 µL of solution from the receiver compartment was removed and replaced by the same volume of PBS. The diffused plasmid into the receiver compartment was evaluated using a microplate reader and compared. Permeation through intact skin was also carried out simultaneously.

### 7.2. In Vivo Studies

The visualization of MNs patches and drug uptake is carried out by an in vivo imaging system (IVIS). It observes the subject animal at a predetermined time interval [215]. Insulin delivery in the induced diabetic Sprague Dawley rats with the help of MNs was conducted [216]. A pneumatically driven insertion device was used for inserting the MNs into the lower back skin of the rat. The MNs were removed with the help of forceps. Around the pierced skin of the rat, a chamber was fixed, which was later filled with Humulin-R insulin (100 u/mL). It was kept for 4 h, and blood glucose measurements were made every 30 min using a human insulin-specific radioimmunoassay. The same protocol was carried out for negative control without MNs insertion in the skin. The positive control, subcutaneous administration of Humulin-R insulin 50 µL diluted with PBS was conducted. The visualization of MNs patch and drug uptake was carried by an in vivo imaging system (IVIS). It observed the subject animal at a predetermined time interval [212]. Figure 14 showed MNs for the delivery of insulin. Here, they found that the insulin that was applied to rats’ skin without a microneedle roller was not found to be significantly different (*p* > 0.05) than the time controlled group, approving in-efficiency of passive insulin absorption through the transdermal route. The subcutaneous administration of insulin rapidly decreased the blood glucose level at 1 h, to around 18%. For the 500 micron group, the glucose level was 18% at 3 h, significantly different from the negative control rats (*p* < 0.05). In comparison to the positive control after 3 h, the changes in blood glucose levels persuaded through the microneedle roller are higher (*p* < 0.05), but there was no significant difference 3 h (initial 3 h). This specifies that microneedle rollers might increase the skin permeability in long-term delivery, which is suitable for the delivery of repeated amounts of pharmacologically active insulin.

## 8. Applications of MNs

The MNs were first introduced for drug delivery applications, and the major objective was the enhanced permeation in the skin using solid and hollow MNs compared to conventional hypodermic needles. The MNs were filled with drug solutions or formulations, or they were coated for improved intradermal drug delivery. Nowadays, MNs are the leading novel technology for several fields of drug delivery, such as intradermal, ocular and intracellular drug delivery. However, the transdermal route is still the leading application area for MNs, especially vaccine-based delivery.

### 8.1. Intradermal Drug Delivery through MNs Formulations

Drug delivery to the skin is challenging as it may be a local application or systemic delivery as a result of the stratum corneum’s highly tough and barrier properties. However, the human stratum corneum thickness is (10–15 µm), and it still prohibits the drugs at therapeutic levels [217]. The Food and Drug Administration approves more than 20 drugs for transdermal patches applications with a molecular weight of less than 400 Da and a higher logP [218]. Due to the challenging barrier of the skin, and despite its few micron sizes, novel MNs formulations were developed to pass the stratum corneum and load the drug into the dermal skin without generating any pain or bleeding in the human host [219]. MNs increased the number of drugs administered through the dermis, considering low molecular weight, biomolecules, vaccines or proteins and other materials [220].

### 8.2. Small Molecules (Low Molecular Weight Drugs)

The small molecule or low molecular weight drugs have higher skin diffusion coefficients than the larger molecule or biomolecule, which could easily penetrate the skin. This is so that the small molecule is quickly delivered into the skin using MNs.

Rojekar et al. have formulated dissolving MNs containing the etravirine and etravirine nanosuspension for long-acting drug delivery and improved HIV infection therapy. They have demonstrated the robust nature of MNs, with significant drug deposition of 12.84 ± 1.33% ex vivo, in neonatal porcine skin for 6 h. The in vivo pharmacokinetic studies demonstrated improved parameters; Cmax exhibited by DMNs containing ETR powder and ETR NS was 158 ± 10 ng/mL and 177 ± 30 ng/mL, respectively. It was also revealed that the improved t1/2, Tmax, and mean residence time (MRT) compared to intravenous ETR solutions indicated the long-acting nature of etravirine delivery using DMNs [220].

Lin Zhu et al. have developed estriol-loaded EMNs to effectively treat radiation-induced injury. For the development of EMNs, biocompatible polymer polyvinyl pyrrolidone K90 was used. The drug is dissolved in methanol and mixed with polymer gel to cast into a mold to obtain the conical-shaped EMNs. The developed EMNs are robust and easily penetrate 200 μm into mouse skin. Most interestingly, these EMNs dissolve very quickly in 5 min, which could help immediately permeate the drug into the skin. The mouse model of the ionizing radiation-induced injury was developed by the source of 6.5 Gy radiation of 60 Co γ ray. Furthermore, EMNs enhanced the peripheral blood leukocytes count in irradiated mice, which protected the bone marrow hematopoietic system and 80% increased the survival rate of the irradiated mice [221].

Alyaa et al. have investigated and developed dissolving MNs using biocompatible and biodegradable polymers, poly(vinylpyrrolidone) (PVP) and hyaluronic acid (HA) to intradermally deliver the Amphotericin-B (AMP-B) to treat the fungal infection. It was found that both polymers used in development reduced the AMP-B cytotoxicity, compared to the drug-free solution. Moreover, it was found that AMP-B-loaded dissolving MNs showed significant antifungal activity compared to plain drugs. Furthermore, MNs maintain the activity of drugs [222].

Ismaiel A. Tekko et al., have for the first time, developed the MNs array patches (MAP) with the higher Cabotegravir or micronized sodium salt loading of (≈3 mg/0.5 cm^2^). The MAP was robust with the skin penetration potential. The tips were dissolved in 30 min, giving immediate deposition of the drug in the skin. Moreover, the in vivo dermatokinetic study in Sprague Dawley rats of both forms of drug-loaded MAP deposited into the skin, forming the depot. Both drug forms are released slowly, maintaining the therapeutic concentrations in the blood for one month for a single application [223].

Alejandro J. Paredes et al. have developed tenofovir alafenamide (TAF) loaded dissolving and implantable MAPs to systematically deliver or release the drug. The developed MNs are mechanically strong enough and could pierce the excised neonatal full-thickness porcine skin and deposit the drug as a depot form. The release study performed using dialysis methods demonstrated the relatively fast drug release in both the formulations. The in vivo studies in rats showed rapid metabolization of the TAF into tenofovir, along with quick elimination of the metabolite from the blood plasma [224].

Maelíosa Crudden et al. have developed Rilpivirine nanosuspension-loaded, dissolving MN array patches (MAPs) for the long-acting delivery in HIV for improved therapy and patient compliance. MAPs were mechanically strong enough to pierce the skin and load the drug as depot formulation for a prolonged effect. In vivo pharmacokinetic studies demonstrated that the mean plasma concentration in rats is 431 ng/mL at seven days, which is about ten-fold larger than the trough concentration found after a single dose administered in the previous clinical studies [225].

Mingshan Li et al. developed a novel strategy for co-formulating the dexamethasone and pro-drug dexamethasone sodium phosphate in the DMNs. That could have led to the immediate effect to achieve long-term drug delivery. The 3D printing technique was used the first time to fabricate the baseplate of the MN. The 3D printed base plate is robust, providing excellent support to the drug encapsulated or loaded tips. These novel trilayer-based MNs have shown the effective drug delivery of dexamethasone, which could be the novel promising drug option for oral and injectable drug delivery [226].

### 8.3. Large Molecules (Biotherapeutics)

Protein and peptides are very unstable and degraded after oral administration. Transdermal drug delivery could avoid this issue; however, delivering all kinds of molecules is difficult due to challenging skin barriers [227]. Using MNs, protein and peptide delivery could be an excellent alternative to the traditional transdermal patches. MNs have excellent mechanical properties as they penetrate the dermis and resolve the problem of penetration and permeation associated with conventional drug delivery. It also has good thermostable properties, which could help in protein and peptide drug delivery [228].

Desmopressin is a synthetic form of vasopressin, the potent peptide hormone used to replace the low vasopressin levels in the therapy. This is used to treat diabetes insipidus, which causes bedwetting in children and hemophilia A. The MNs formulation is a novel approach to deliver the desmopressin, which showed an effective and safe delivery compared to the other conventional routes [14].

Liu et al. have formulated GAP-26, a gap-junction blocker containing polyethylene glycol diacrylate MNs to deliver peptides by swelling effect. The developed MNs formulation has improved the permeation of peptides, which leads to improved inhibition of the keloid fibroblasts and the collagen I expression [14,229]. Cyclosporin A is a high molecular weight, a hydrophobic molecule with a cyclic peptide used to treat several skin and dermal diseases. Cyclosporine A loaded dissolving MNs was developed with 600 μm in length, and 250 μm wide was prepared by a molding process. This fabricated MNs formulation delivered 10% *w*/*w* of Cyclosporin A in the porcine skin for 60 min. Approximately 65% of MNs were dissolved with a 34 ± 6.5 μg drug delivery [14,230].

Insulin is the hormone for modulating blood glucose levels with a 51-amino-acid peptide. However, the exceptionally high pain triggered by frequent subcutaneous injections could adversely affect the patient’s compliance [228,231]. Despite this, transdermal delivery of insulin is an eye-catching delivery method. With SC injections, MN-loaded insulin delivery would benefit diabetic patients through self-administration and low pain. The solid MNs fabricated by diverse materials, such as polymer, silicon and metal, have effectively decreased the blood glucose level by improving the insulin permeability by skin pre-treatment [232].

Zhou et al. demonstrated the applicability of the stainless steel MNs with different needle lengths, which were used to evaluate the delivery efficacy of insulin in diabetic rats. The results demonstrated that the skin permeability increased significantly with a rapidly decreasing glucose level within 1 h of application. It is also seen that solid MNs associated with the iontophoresis could effectively improve the intradermal delivery of the insulin [233]. McAllister et al. demonstrated that hollow MNs can deliver the microliter solution to the skin; however, larger pressure could trigger a faster decrease in blood glucose levels. The hollow MNs-based intradermal insulin delivery resulted in faster insulin onset, driven by the passive diffusion, electricity or pressure [232]. Li et al. have optimized and developed MNs to study the effect of insulin delivery on blood levels in mice. It was found that blood glucose level was decreased to 29% of the initial level at 5 h, which could confirm the improved insulin permeability using MN-based drug delivery [14,109].

**Figure 15:**
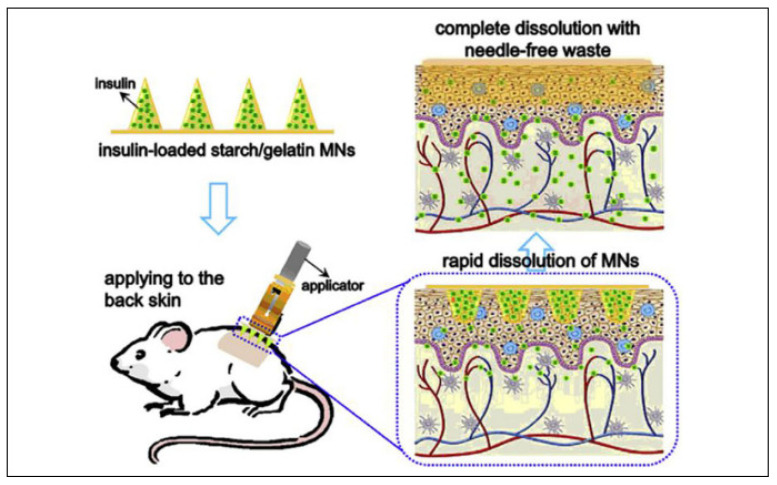
MNs for insulin delivery [237] (Reprinted with permission from Ref. [237]. Copyright 2013 Elsevier).

**Figure 16:**
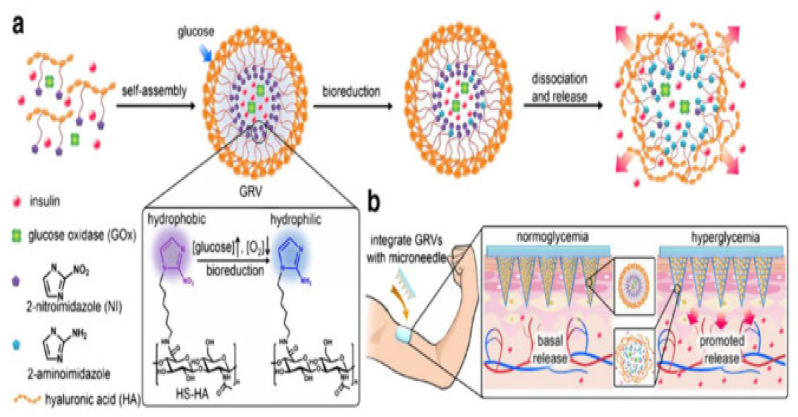
MNs for delivery of insulin (**a**) Formation and mechanism of GRVs composed of HS-HA. (**b**) Schematic of the GRV-containing MN-array patch (smart insulin patch) for in vivo insulin delivery triggered by a hyperglycemic state to release more insulin [238]. (Adapted from Ref. [238]).

Ye and co-workers have studied MNs association with pancreatic β-cell capsules, which could sense the glucose level in the blood and secrete insulin as per the requirements. It was found that the patch was not effective enough. MNs matrix-loaded synthetic glucose signal amplifiers were developed. These MNs include α- amylase, glucoamylase, and glucose oxidase; this indicates insulin secretion from the β-cells capsules [14,234]. The clinical study of the parathyroid hormone (I-34) coated MNs demonstrated two times shorter t_1/2_ and three times shorter Tmax than the conventional injectable therapy [235]. These studies demonstrated the MN’s potential capability in hormonal drug delivery, suggesting MNs formulations’ effectivity and efficiency. These could also be altered for sustained effect by using appropriate polymers. Furthermore, iontophoresis united with MNs could also be discovered to deliver numerous hormones [14,236]. Figure 15 and Figure 16 showed MNs for delivery of insulin.

### 8.4. Other Biomolecules

The DNA and RNA are short oligonucleotides, basically smaller units than proteins. Delivery of the oligonucleotide is difficult due to their properties, so numerous techniques were employed to deliver these agents. The delivery of the 20-merphosphorothioated oligodeoxynucleotide was conducted using the MNs formulations approach. The solid MNs, made from stainless steel, were used to deliver these oligonucleotides via the poke with the patch approach. It was found that more drugs were delivered using this approach compared to intact skin [14,239].

### 8.5. Vaccine

**Figure 17:**
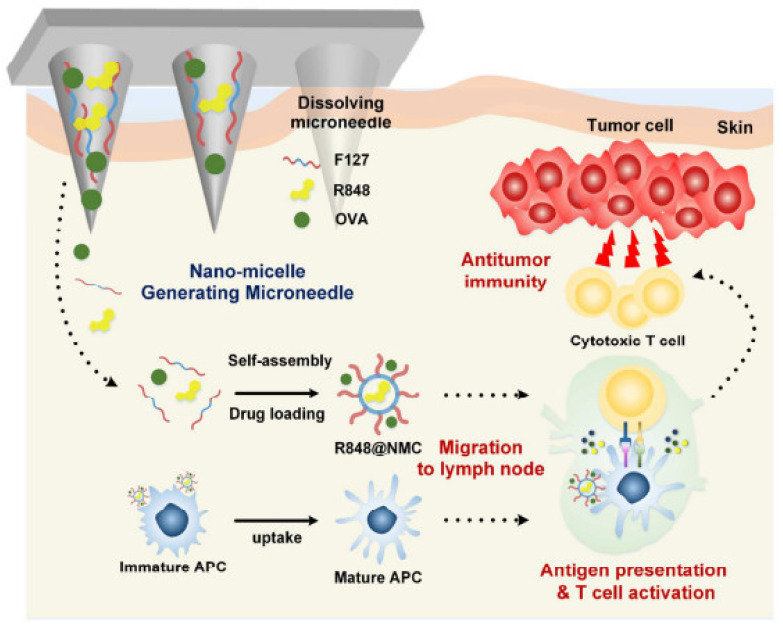
Dissolving MNs loaded with vaccines and hydrophobic adjuvants for improved cancer therapy [244]. (Adapted with permission from Ref. [244]. Copyright 2018 American Chemical Society).

A vaccine is a complex biological preparation or formulation. It successfully offers active acquired immunity to a specific disease. Vaccines consist of the killed or weakened form of disease triggering microorganisms, toxins or one of its surface proteins [109]. Vaccines could stimulate the body’s immune system and protect the host system against future infections or diseases [240]. The MN-based intradermal vaccine drug delivery was an excellent and effective option. The MNs have delivered the DNA-based vaccine and have obtained immune responses that were much better than regular injections [241]. An attempt to develop an MNs patch to administer the influenza vaccine was made [242]. A lower dose is required when the drug is administered using hollow MNs compared to when using an intramuscular injection. Anthrax and rabies vaccine delivery using hollow MNs have been studied [3]. Ogai et al. have developed hollow MNs by using biodegradable PLGA to improve the delivery and efficiency of the vaccine by intradermal route. It was demonstrated that the drug or vaccine delivery in the upper dermis could provide improved immunity. Furthermore, it was found that the antibody titers were significantly higher than conventional delivery [230,243]. Figure 17 showed Dissolving MNs loaded with vaccines and hydrophobic adjuvants for improved cancer therapy.

### 8.6. Diagnosis

The painless withdrawal of the biological fluids from the body is the major advantage of MNs over conventional blood collection techniques. Various biomarkers present in interstitial fluid beneath the skin can be useful for diagnosing various diseases like diabetes, cancer, arthritis, etc., and helpful, timely medical intervention. Multiple research studies demonstrated the usefulness of MNs for disease diagnosis [245]. Chang et al. revealed the application of MNs in the extraction of interstitial fluid to analyze metabolites. The MNs patch consisting of methacrylate hyaluronic acid efficiently extracted skin interstitial fluid. The extracted fluid can be further used for diagnostic purposes [246]. Jin and coworkers reported the usefulness of MNs in Tuberculosis (TB) skin tests (Mantoux test). The MNs efficiently deliver the purified protein derivative (PPD) in TB skin tests. The precise and controlled delivery in deep skin is advantageous for using MNs [247]. El-Laboudi et al. stated the effective use of MNs array in monitoring glucose, which could be useful in diagnosing diabetes and associated disorders [248]. Pires et al. described the use of MNs in pediatrics. Vital vaccination like tetanus, diphtheria, and pertussis is carried out within a year from the child’s birth. The use of MNs can help overcome the discomfort and phobia associated with conventional needles and results in efficient child vaccination.

Along with vaccination, the MNs can be used to diagnose various skin diseases in pediatrics like psoriasis and other inflammatory conditions [249]. One of the prime applications of MNs is in diagnosing various carcinogenic conditions. Multiple anticancer vaccines and drugs are delivered through MNs using nanocarriers. The delivery of these cancer diagnostic agents through MNs shows improved biodistribution and efficient diagnosis [250].

### 8.7. Biosensing

**Figure 18:**
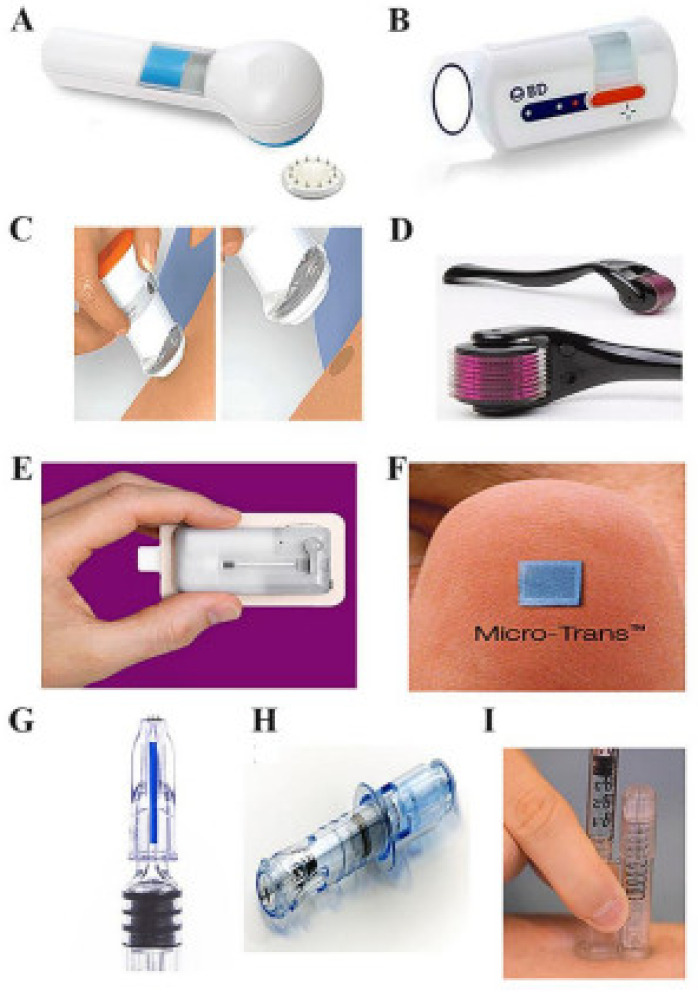
Commercially available MNs devices (**A**) Microstructured Transdermal Syst **(B)** BD Microinfuser^®^ (**C**) MicrofluxrTM (**D**) MTS RollerTM (**E**) Vaaleritas h-patchTM (**F**) MicrotransTM (**G**) MicronJet^®^ (**H**) Intanza^®^ (**I**) DebioJectTM [259]. (Reprinted with permission from Ref. [259]. Copyright 2019 Elsevier).

MNs provide significant efficiency in the biosensing of various biomarkers and metabolites. Collection of biofluid is more convenient with MNs than with conventional hypodermic needles. Recent advancements in MNs are resulting in improved biosensing [251]. Strambini et al. demonstrated MN-based biosensors to detect glycemia in interstitial fluid [252]. Electrochemical biosensors are emerging advancements in MNs technology used for biosensing. Innovation geometrical configurations of MNs provide an advantage in biosensing [253]. After their research, Zho et al. reported that the MNs fabricated from silk, polyols, and glucose oxidase could be used for glucose biosensing by electrochemical biosensing technology [254]. Bollela and coworkers reported the development of second-generation MN-based biosensors to detect lactate. The gold MNs were functionalized with nano carbons, through which the electron transfer of lactate oxidase took place, resulting in efficient sensing of lactate [255]. Polymer-based MNs are also frequently used for biosensing. Multiple researchers across the globe have reported the usefulness of polymeric needle-based MNs in biosensing of various endogenous substances [256]. Calio et al. demonstrated the use of MNs fabricated from poly (ethylene glycol) diacrylate in biosensing applications. The prepared polymeric MNs were used to fabricate the electrodes used for biosensing of glucose and lactic acid [257]. Various carbon-based MNs are also used in biosensing. Jin et al. demonstrated the usefulness of hybrid MNs consisting of reduced graphene oxide and platinum nanoparticles for the biosensing of hydrogen peroxide. The in vivo study on pig skin and living mice proved the biosensing efficiency of prepared hybrid MNs [258]. Figure 18 shows commercially available MNs devices.

### 8.8. Cancer Therapy

**Figure 19:**
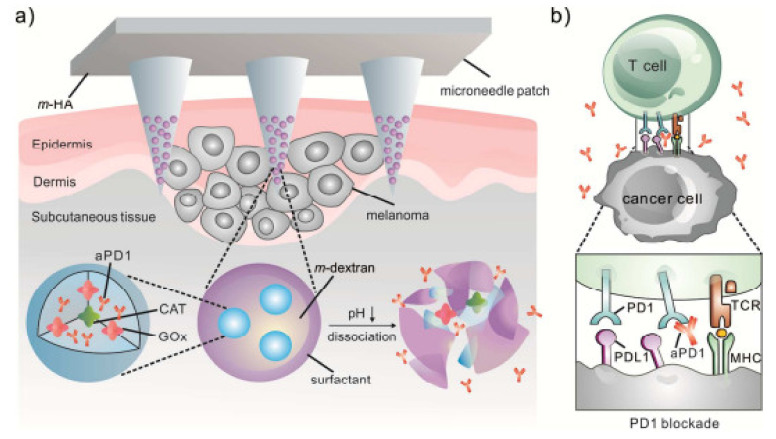
Biodegradable Hyaluronic acid MNs (HAMN) containing antibodies for the treatment of skin cancers. (**a**) Schematic of the aPD1 delivered by an MN patch loaded with physiologically self-dissociated NPs. (**b**) The blockade of PD-1 by aPD1 to activate the immune system to destroy skin cancer cells. [263]. (Reprinted with permission from Ref. [263]. Copyright 2016 American Chemical Society).

MNs technology creates a new horizon in cancer therapy through efficient drug delivery of anticancer vaccines and drugs. Various chemotherapeutic agents, genes and proteins can be efficiently delivered through MN-based devices [260]. Hao et al. established the usefulness of MNs technology in the treatment of epidermoid cancer therapy. The researchers developed the near-infrared responsive PEGylated gold nanorod and Doxorubicin-containing dissolvable Hyaluronic Acid MNs for the localized efficient therapy of epidermoid cancer, and it showed efficient antitumor activity [261]. Gadag and coworkers also demonstrated the usefulness of MNs in breast cancer therapy. Resveratrol is one of the efficient anticancer agents used to treat breast cancer but is limited by low bioavailability. To overcome this limitation, researchers developed the nanostructured lipid carriers of Resveratrol and delivered them through MNs arrays. The drug delivery through MNs improved permeation and bioavailability at the tumor site [262]. Hao et al. demonstrated the application of MNs in skin cancer therapy. The researchers fabricated the near-infrared responsive 5-indocyanine green and fluorouracil containing monomethoxy-poly (ethylene glycol)-polycaprolactone nanoparticles delivered through dissolvable MNs efficient therapy of human epidermoid cancer and melanoma [93]. Moreira et al. demonstrated the efficient delivery of doxorubicin and AuMSS nanorods through polyvinyl alcohol/chitosan layer-by-layer MNs, resulting in efficient cancer chemo-photothermal therapy [104]. Lan and coworkers also provided MN’s significant applications in delivery proteins in cancer immunotherapy. They fabricated an MNs patch containing Ph responsive tumor-targeted lipid nanoparticles loaded with PD-1 and cisplatin, resulting from precise drug delivery and efficient immunotherapy [99]. Figure 19 showed Biodegradable Hyaluronic acid MNs (HAMN) containing antibodies for the treatment of skin cancers.

### 8.9. Ocular Drug Delivery

Bypassing the ocular barrier with minimum invasion is the advantage of MNs over intravitreal injection. Several studies demonstrated the application of MNs in ocular drug delivery. Patel et al. demonstrated the successful delivery of micro and nanoparticle suspension in the suprachoroidal space of pig, rabbit and human eye (ex vivo) using hollow MNs. Optimizing dimension and process parameters concluded that efficient drug delivery could be achieved with a needle length of 800–1000 µm and pressure of 250–300 kPa [264]. Through their research, Jiang et al. also endorsed the application of MNs technology in ocular drug delivery. Using the coated solid MNs, the intrascleral and intracorneal delivery of drugs, protein, and DNA was assessed. The successful delivery of drugs in the ocular system with minimum invasion was observed [265].

## 9. Toxicity Study Methods

The toxicity of MNs is checked by examining the materials used in their fabrication, such as silicon and stainless steel, and polymers, i.e., poly-methyl methacrylate, poly-lactic acid, etc., cause the toxicity of MNs. Sometimes the presence of materials such as photoinitiators can result in the toxic effects of MNs [227].

### 9.1. In Vitro Method

In vitro methods for toxicity assessment of MNs are carried out using cell line assays. Commonly used cell lines are human adult low calcium high temperature (HaCaT) keratinocytes and human embryonic kidney (HEK293) [210]. Piu et al. have carried out the cytotoxicity assay by cell viability for cellulose acetate MNs. NIH-3T3 fibroblasts cells were used for the study. The porous patches made up of polymers cellulose acetate, polysulfone, and polyethersulfone were sterilized with alcohol and soaked with PBS in triplicate to remove the alcohol. Uv irradiation was conducted for 2 h. The fibroblast cells were seeded into 48-well plates, with a cell density of 1 × 10^4^ per well. The incubation media used was 90% of DMEM and 10% of fetal bovine serum. The sterilized MN patches were put into the wells, and this was kept for incubation for 24 h. Untreated cells were used as control. For analysis, 100 µL of culture media from each well was transferred to a 96-well plate, and the absorbance was checked with a microplate reader at 450 nm [266]. In another study on PEGDA MNs, Human vein endothelial cells (HUVECs) are used. The HUVECs are cultured in 96-well plates with a seeding density of 1 × 10^4^ for 12 h. The MNs patch of 0.5 cm^2^ was soaked in 1 mL DMEM media for 24 h and this was used as a soaking solution. Next, 200 µL of soaking solution was poured to each well and incubated for 24 h. Supernatant was replaced by 200 MTT µL agent. Then, 200 µL of DMSO was added to each plate and gently shaken for 15 min. The absorbance was checked at 570 and 630 nm and relative cell viability was calculated [267].

### 9.2. In Vivo Method

Biocompatibility of MNs by in vivo methods is usually assessed by acute dermal toxicity study. An MNs patch is placed on to the shaved skin of rats and observed visually to see whether the inflammation has occurred or not [268]. The material used in the fabrication of MNs is also checked for biocompatibility study with the help of a cell lines study. The cell lines used are the human dermal fibroblasts, HaCaT, and HEK293.

## 10. Regulatory Aspects

For genuine MNs goods, there are currently no agreed regulatory requirements. Precise quality requirements in the framework of Good Manufacturing Practice need to be developed for MNs to be made on an industrial scale. Currently, standardized testing and equipment utilized to validate MNs mechanical characteristics and insertion capacity are missing, making it hard to compare MNs and necessitating the adoption of standardized tests and equipment to evaluate product quality and adequate least requirements. There is the need to perfect the implementation of a pharmaceutical quality system with good manufacturing practice and quality risk management for well-designed MNs to commercialization. A stringent rule and regulation should be applied to meet all drug product requirements before being released for human use. The complex regulatory specifications and time requirements will impact the cost of the end finished product [269,270]. The Food and Drug Administration (FDA or Agency) published a draught guideline titled “Regulatory considerations for micro-needling devices: draught guidance for industry and FDA staff” on 15 September 2017. In the current circumstances, this guideline has not been finalized or applied. However, the guidance is very informative and provides sufficient knowledge, but more test data is needed to investigate the clinical applications of MNs. As a result, data on safety is important. Short-term and long-term safety data should be collected more precisely and effectively to enable the use of MNs devices in today’s environment [44]. Table 3 shows the marketed product for MNs for different applications.

## 11. Patents

MNs are a new approach to administered medications via the stratum corneum [144]. MNs are well-known for their efficiency, and they have been widely employed in the delivery of insulin, biological macromolecules, and vaccinations. Nevertheless, their minimal invasiveness, stability concerns, and non-compliance are a source of concern, necessitating highly competent medical practitioners to administer them [274]. MNs have shown their potential in this context since they can administer medications with ease, painlessly, and safely without specialized storage conditions. Here, some patents are briefed, which summarize its scope. Table 4 shows recent patents on MNs for different applications.

## 12. Conclusions

This review article extensively describes the type of microneedle, fabrication material, detailed casting methods and techniques, and its applications. Microneedles have been fabricated using different materials, like silicon, metals, polymers, and ceramics, by several fabrication methods, i.e., lithography, wet and dry etching, laser cutting and micro molding. The practical use of microneedles has been acknowledged and gained widespread attention. Optimization of sharpness, length, insertion force and velocity, and other parameters have allowed reliable microneedle insertion into the skin. Nowadays, MNs are the leading novel technology for several fields of drug delivery, such as intradermal, ocular and intracellular drug delivery. However, the transdermal route is still the leading application area for MNs, especially vaccine-based delivery. MNs technology creates a new horizon in cancer therapy through efficient drug delivery of anticancer vaccines and drugs. Various chemotherapeutic agents, genes and proteins can be efficiently delivered through MN-based devices. Patients and clinical workers are highly inclined to prefer microneedle-based delivery over hypodermic injections according to surveys. Human subjects report little or no pain associated with most microneedle designs. After microneedle treatment, the skin often shows mild, transient erythema, but there is currently no evidence of increased infection risk at the treatment site. Big pharmaceutical giants are currently working on the developments and commercialization of microneedle-based drug delivery systems; this technology is rated in the top 10 recent technologies. Patients, healthcare providers and companies have established interest in the technology. Microneedles are poised to make an expanded impact on clinical medicine over the coming years.

## 13. Future Scope

Microneedles are being studied worldwide as a drug delivery system for various ailments, including treatment of all diseases and vaccination. The development of sophisticated delivery systems like MNs could boost the efficiency of drug administration and lower the total dose concentration to minimize side effects. Some characteristics, such as the needle dissolution rate, can affect bioavailability. As a result, changing the needle geometry and size could assist us in achieving a more profound transdermal drug release. Novel MNs production technologies have resulted from much research in this field. MNs devices improve patient compliance by avoiding site-specific infections caused by conventional needles. However, the discovery of innovative micro-fabrication processes and stability medicines for MNs devices remains a challenge. Some of the primary reasons driving the market expansion include the increased prevalence of chronic hyperpigmentation and skin infections worldwide. Another factor driving the market over the forecast period is the low cost of microneedle devices compared to plastic surgery. MNs are also anticipated to be a revolutionary tool in the Cosmetic Industry. Furthermore, changing lifestyles are predicted to increase skin infections, and the availability of at-home micro-needling devices is expected to drive market expansion. Lack of experience, lack of public consciousness, and treatment side effects are some of the limitations on market growth.

## Figures and Tables

**Table 2:** Material used for fabrication MNs.

Material Used for MNs	Material Used in a Publication (in %)
Metal	14
Glass	5
Ceramic	3
Silicon	10
Polymer	68

**Table 3:** Marketed product for MNs.

Product Name	Company	Approved for Condition	Description	Reference
Fluzone	Sanofi Pasteur Inc.	Influenza Virus Vaccine (USFDA)	Micro-injection system for intradermal delivery of vaccine	[271]
Intanza	Sanofi Pasteur Europe	Split virion, Inactivated Influenza vaccine (EMA) (Discontinued due to commercial issues)	Micro-injection system of a prefilled syringe, having a 1.5 mm needle length. The needle shielding system is provided, which covers the needle after use.	[272]
C-8 (Cosmetic type)	The Dermaroller Series by Anastassakis K	Cosmetic use.	It has a 0.13 mm needle length. It enhances the penetration of topical agents	[273]
C-8HE (Hair-bearing surface)		Cosmetic Use	It has a 0.2 mm. needle length. The length is below the pain threshold, so painless delivery	[273]
CIT-8 (Collagen Induction Therapy)		Medical type	It is used for collagen induction and skin remodeling therapy. It has a 0.5 mm needle length.	[273]
MF-8		Creating deeper microchannels on the epidermis	Needle length of 1.5 mm, its deep penetration is targeted for destroying bundles of scar collagen	[273]
MS-4		Facial acne scars	The needles have 1.5 mm in length and 4 circular arrays. Its use is preferred where better precision and penetration in deep location is required.	[273]

**Table 4:** Recent patents on MNs.

Patent No.	Title of Patent	Aim	Description
US 10,898,703 B2	MNs template and MNs prepared using the same.	Preparation of MNs template; Preparation of MNs, using a prepared template and MNs preparation method.	An MNs template includes a substrate on which a minimum of one MNs shape is projected to which at least one diamond layer is formed on the MNs surface.
US 2021/0008360 A1	Adhesion membrane and MNs patch	To provide a new patch with excellent flexibility to skin, which carries an MNs array patch.	MNs provide excellent skin punctures and also stay on the skin; Provides variation in puncture property depending on the elasticity of the skin.
US 2021/0030975 A1	Application for applying an MNs device to the skin	An applicator, method for application of MNs device to the skin	Applying the MNs device on the skin delivers active ingredients for treatment using applicators and methods.
US 10,946,180 B2	Applicators for MNs	Description of micro projection array for MNs application	Applicator and method for applying MNs for treatment.
US 10,973,757 B2	Biodegradable MNs device	To provide one or more biodegradable MNs capable of drug administration to the skin.	The invented device is embodied in MNs form for skin applications. At least one biodegradable MNs is projected from the cap with the lower surface abutted. MN projection is formed by a polymeric blend of preserved stem cell factors.
US2021/0046299A1	Composite MNs array including nanostructures thereon	A composite MNs array overlays the film (consists of a plurality of nano-sized structures fabricated thereon) with MNs.	MNs array and MNs assembly and film consist of a plurality of nano-sized structures fabricated for drug delivery applications.
US 2021/0106520 A1	Conductive polymer MNs arrays for electronically controlled drug release	A method that delivers a therapeutic agent, provided with an MNs array, also includes a plurality of MNs, including conductive coating disposal.	Conductive coating derived controlled therapeutic agent release by using MNs array implant in and across the dura mater to CNS of the subject.
US 10,987, 503 B2	Dissolvable MNs for skin treatment	A skin treatment includes MNs application on the skin and penetration to the stratum corneum.	Polymeric MNs and their methods as a skin treatment device
US 10,994,111 B2	Drug holding MNs array and manufacturing method thereof	To provide a drug-holding MNs array, where the drug is applied and held on the area of the tip of the MNs, for holding the dose capacity and prevention of drug spillage during insertion.	Technique for drug holding into MNs by step formation on MNs for quantitative dose administration
US 2021/0106259 A1	The electrically functional polymer MN array	A sensor (biosensor) device comprises a polysubstrate substance structured to form MNs.	A device with electrodes, related devices, apparatus and fabrication methods, and devices use
US8708966B2	MNs devices and methods of manufacture and use thereof	A method for delivering the active agent across a biological barrier	Devices with MNs are available for transporting compounds across tissue barriers and serving as microflameholders

## Data Availability

This research did not report any data.
